# High‐throughput metabolomics predicts drug–target relationships for eukaryotic proteins

**DOI:** 10.15252/msb.202110767

**Published:** 2022-02-23

**Authors:** Duncan Holbrook‐Smith, Stephan Durot, Uwe Sauer

**Affiliations:** ^1^ Institute of Molecular Systems Biology ETH Zurich Zurich Switzerland

**Keywords:** chemical screening, drug–targets, metabolomics, overexpression, signaling, Metabolism, Methods & Resources, Pharmacology & Drug Discovery

## Abstract

Chemical probes are important tools for understanding biological systems. However, because of the huge combinatorial space of targets and potential compounds, traditional chemical screens cannot be applied systematically to find probes for all possible druggable targets. Here, we demonstrate a novel concept for overcoming this challenge by leveraging high‐throughput metabolomics and overexpression to predict drug–target interactions. The metabolome profiles of yeast treated with 1,280 compounds from a chemical library were collected and compared with those of inducible yeast membrane protein overexpression strains. By matching metabolome profiles, we predicted which small molecules targeted which signaling systems and recovered known interactions. Drug–target predictions were generated across the 86 genes studied, including for difficult to study membrane proteins. A subset of those predictions were tested and validated, including the novel targeting of GPR1 signaling by ibuprofen. These results demonstrate the feasibility of predicting drug–target relationships for eukaryotic proteins using high‐throughput metabolomics.

## Graphical abstract

High‐throughput metabolome profiling identifies potential drug‐target relationships by comparing drug‐treated and induced overexpression *S. cerevisiae* strains. The approach retrieves known as well as new potential drug‐gene relationships.

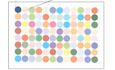

## Introduction

Chemical screening refers to a broad array of experimental techniques where the biological or biophysical properties of collections of small molecules are assayed in order to identify small molecules with some activity of interest (Entzeroth *et al*, 2009). This family of approaches has long been a source of chemical probes that can be used to understand signaling systems by selectively and dynamically modulating their activity (Zeng *et al*, 2005; Filippakopoulos & Knapp, 2014), as well as a source of lead compounds for pharmaceutical development (Swinney, 2013). In a classical target‐based chemical screen, one macromolecular target is chosen and an assay is devised that can monitor the ability of small molecules to perturb the target. Although this general approach has proven useful in many instances (Capdeville *et al*, 2002; Wakeling *et al*, 2002), for any additional protein that becomes a target the screening process must be started anew. If we are to be able to systematically identify chemical probes and use them as tools to understand cellular signaling, a method for reducing the combinatorial space of thousands of druggable macromolecules (Hopkins & Groom, 2002) and drugs is needed. One way to reach this goal is through the prediction of drug–target relationships. A range of approaches has been used in the past to predict drug–target relationships. These include computational docking of small molecules in known structures (Kuntz *et al*, 1982), structure‐independent machine‐learning based strategies (Erhan *et al*, 2006), among many other approaches (Hieronymus *et al*, 2006; Carpenter, 2007; Gregori‐Puigjané *et al*, 2012; Feng *et al*, 2014). These approaches have strengths and weaknesses (Scior *et al*, 2012), but none of them currently appears to hold the promise of universal prediction of drug–target interactions.

An experimental alternative to prediction could exploit that genetic mutations and small molecule drugs alike are able to alter the activity of their target macromolecules and hence might cause similar phenotypes (Baum *et al*, 2010; Campos & Zampieri, 2019). Thus, it can be possible to predict which drugs alter the activities of which gene products or processes based on the similarity of the phenotypes they elicit (Stegmaier *et al*, 2004). However, because traditional chemical screens have only allowed for the measurement of relatively small numbers of parameters for each drug treatment, phenotypic data generated in chemical screens have not contained enough information to predict drug–target interactions. Marshalling omics approaches into chemical screening offers opportunities to overcome this limitation. Transcriptional, proteomic, or metabolomic profiles can serve as a fingerprint of the state of the cell (Raamsdonk *et al*, 2001) and allow for the comparison of various states in order to generate predictions of mode of action (Zampieri *et al*, 2018). Although transcriptomics and proteomics can offer fine grained and gene‐specific representations of the cellular state, with current technologies both approaches cannot offer the throughput at a reasonable cost that is necessary for large‐scale chemical screening. By comparison, with the advent of metabolomics techniques where the measurement time per sample is < 1 min (Fuhrer *et al*, 2011), it is now possible to measure the effects of thousands of drug‐like molecules on the relative abundances of metabolites in the cell. The application of metabolomics approaches to *Saccharomyces cerevisiae* has allowed for powerful investigations of gene function (Allen *et al*, 2003; Zhu *et al*, 2012; Breunig *et al*, 2014) as well as broader questions of how growth is coordinated (Kresnowati *et al*, 2006; Castrillo *et al*, 2007).

Here, we apply metabolomics approaches to *S. cerevisiae* in order to demonstrate proof of concept that it is possible to predict drug–target relationships by comparing the metabolome profiles of inducible overexpression strains to yeast treated with 1,280 drugs from a chemical library. Since membrane‐bound proteins are an important and challenging target class, we first decided to focus on a collection of 6 inducible overexpression mutants in membrane‐bound receptors. This approach was shown to be able to recover known as well as novel relationships between drugs and signaling systems. We subsequently expanded our study to 80 more proteins that are both intracellular and membrane bound in such a way that could be employed at a genome‐wide scale. That approach was used to successfully recapture known drug–target interactions for both membrane‐bound and cytosolic targets, for both signaling and metabolic targets, suggesting that this approach has broad potential to predict drug–target interactions in yeast and perhaps eventually in mammalian systems.

## Results

### Metabolome profiling of inducible overexpression receptor mutants

**Figure 1:**
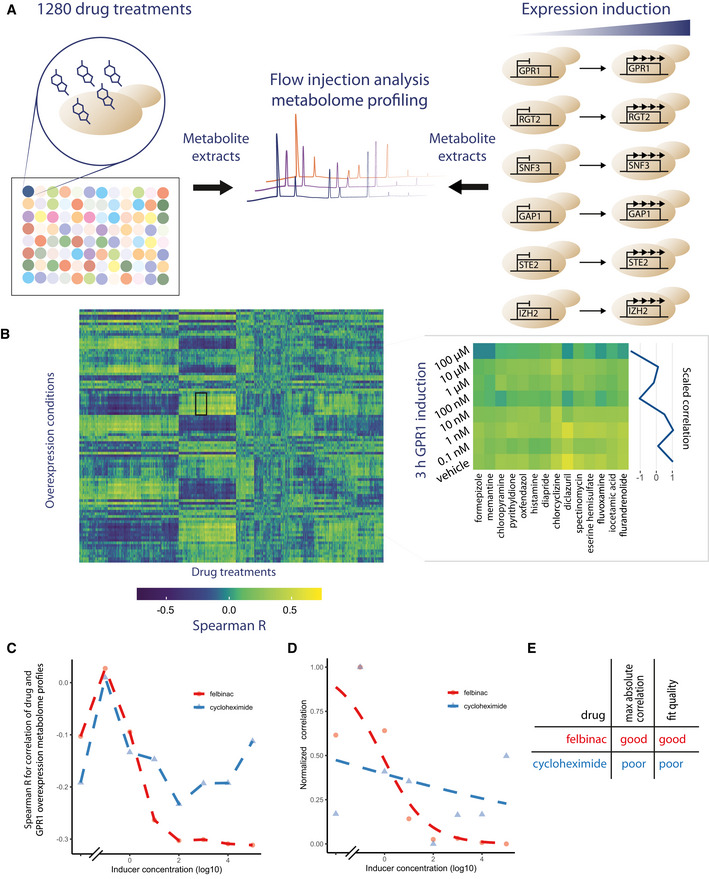
Method schematic for drug–target prediction through metabolome profiling Schematic diagram of study organization. Metabolome profiles were collected from yeast that were treated with 1,280 drugs as well as yeast where 6 genes were independently expressed.The Spearman correlation coefficients for the comparison of *z*‐scored drug and overexpression metabolome profiles are shown in a heat map format with a call out of the indicated cluster. In that cluster, a pattern of decreasing correlation was observed as is indicated by the trend in average −1 to 1 scaled correlations shown in the line plot.The relationship between the Spearman correlation of the metabolome profiles of yeast treated with drugs and each GPR1 overexpression condition is shown for two example drugs across the different concentrations of inducer that were used.The fit of a logistic curve to the normalized correlation data (scaled between 0 and 1 for minimum and maximum values) from C is shown.A summary of the analysis of the two example drugs.

To elucidate whether it is possible to predict drug–target interactions based on metabolome profiles of overexpression mutants in a eukaryotic context, six membrane protein encoding genes from yeast were targeted for analysis (Fig 1A). Three of them (GAP1, RGT2, and SNF3) coding for transceptors (Conrad *et al*, 2014), two encode G‐protein coupled receptors (GPR1, and STE2) (Versele *et al*, 2001), and one encodes the thaumatin receptor IZH2 (Villa *et al*, 2009). The chosen β‐estradiol inducible overexpression system offers several benefits including high overexpression levels, being based in a prototrophic strain background, and tight control over gene expression (McIsaac *et al*, 2013). Since the native promoter is replaced by a tight synthetic promoter, in the absence of inducer the phenotype of the overexpression strains generally reflect a loss of expression of the target gene (McIsaac *et al*, 2013), although some low copy genes appear to be expressed sufficiently in the absence of estradiol for some level of function (Arita *et al*, 2021). The tunable nature of the overexpression system was exploited by assessing the metabolic effect of treating the strains with a wide range of inducer concentrations as well as two time points.

Yeast were cultivated in 96‐well format deep well plates in synthetic defined media with amino acids and were grown from a starting optical density at 600 nm (OD_600_) of 0.1. Eight different concentrations of the inducer, spanning seven orders of magnitude, or DMSO controls were added for a duration of 1.5 and 3 h. These conditions were chosen to allow for robust expression of the receptors without necessarily allowing the cell time to compensate for their expression. DMSO concentrations were kept consistent across all treatments here and in subsequent experiments so that the effect of the solvent on the metabolome (Allen *et al*, 2004) was held constant. The treatments were timed such that a final OD_600_ of ~1.0 was achieved at the time that metabolite extracts were collected. Cells were harvested by centrifugation and metabolites were extracted with cold solvent from cell pellets. The intracellular metabolite extracts were analyzed by flow‐injection analysis time‐of‐flight mass spectrometry (FIA TOF MS) (Fuhrer *et al*, 2011). FIA TOF MS makes use of a chromatography‐free system that allows for high‐throughput profiling of metabolite extracts. The tradeoff for this throughput lies in the inability of the system to resolve compounds with the same molecular weight, and that it can misannotate ions or misquantify the abundance of ions that are in crowded regions of the mass spectrum. The 13,615 ions were identified after spectral processing, of which 1,029 could be annotated to a compound within the KEGG compound library. Analysis was restricted to the 226 metabolites that are part of the *S. cerevisiae* KEGG collection. Raw ion intensities were normalized for temporal drifts and also for biomass at the time of sampling. Although growth rate is known to exert a strong effect on the metabolome (Castrillo *et al*, 2007; Boer *et al*, 2010; Campos & Zampieri, 2019), after biomass normalization there was no appreciable increase in hit quality from growth rate normalization and so it was omitted. This resulted in the observation of robust changes in the metabolome, where 37% of overexpression treatments caused at least one metabolite to change in abundance by at least two‐fold, and the median maximum absolute log2 fold change observed for each treatment was 0.9. Dose‐dependent patterns of metabolic changes were seen for the different concentrations of inducer added. For example, disaccharides accumulated in GPR1 overexpression strains in conditions where < 1 nM of inducer was added, and a decrease in disaccharide levels occurred in cases where more inducer was added (Appendix Fig S1). The accumulation of the disaccharide trehalose is a previously observed phenotype of GPR1 loss of function mutants (Iyer *et al*, 2008) that occurs due to its contribution to signaling through protein kinase A (Kraakman *et al*, 1999). These results show that the range of inducer concentrations used is sufficient to generate overexpression that causes measureable effects on the metabolome. This is a necessary precondition for a meaningful comparison of metabolome profiles between overexpression and drug treatments with the goal of matching profiles to predict drug–target relationships. In addition, the abundance of amino acids also increased in abundance under the induced overexpression of the amino acid transceptor GAP1 (Appendix Fig S1). The induced overexpression of the other targets also showed altered patterns of abundance of metabolites in central metabolism such as hydroxyethyl thiamine diphosphate for SNF3 and oxoglutarate for RGT2 (Appendix Fig S1).

### Metabolome profiling of yeast treated with chemical library

Similar to the overexpression experiments, yeast were cultivated and drug treatments were timed such that cultures grew exponentially in synthetic defined media with amino acids from a starting OD_600_ of ~0.1 to an OD_600_ of 1.0 at the time of metabolite extraction, placing them within an exponential growth phase. Preliminary experiments showed that drug treatments could cause significant metabolome changes within 30 min (Appendix Fig S2), and the relatively short treatment had the advantage of capturing the effects of drugs on the metabolome before extensive acclimatization or effects on growth rate has occurred. Within the main screen, the entire Prestwick chemical library of 1,280 FDA‐approved compounds was applied to the same background strain of yeast. The drug treatments were performed at a concentration of 10 µM and metabolite extracts were prepared and analyzed using the same FIA TOF MS approach described earlier. Despite few of the drugs having a known target in *S. cerevisiae*, 14% of drug treatments caused at least 1 of the 226 annotated metabolites to increase or decrease two‐fold compared with the batch average value, and the median drug treatment had a maximum metabolite log2 fold change of 0.83. This demonstrates that in general the drug treatments were able to perturb the yeast metabolome. Results exhibited several expected metabolome changes for drugs with known targets within yeast. For example, the protein synthesis inhibitor cycloheximide showed an increase in the abundance of many amino acids, consistent with previous reports (Mülleder *et al*, 2016) (Appendix Fig S3A and B). Statins also showed an accumulation of HMG‐CoA (Appendix Fig S3C) that is likewise consistent with its role as an HMG‐CoA reductase inhibitor that is active in yeast (Callegari *et al*, 2010).

### Comparison of drug and overexpression metabolome data

In order to identify which drug treatments elicit similar metabolome profiles to those of the inducible overexpression mutants, the Spearman correlation coefficients for each overexpression mutant with each drug treatment were calculated for both time points (Fig 1B). Within these correlations, it was possible to identify cases where there was a trend with respect to the degree of similarity of metabolite profiles in a drug and overexpression condition (Fig 1B right panel, Fig 1C). To systematically identify and assess the quality of the relationships between overexpression strength and metabolome profile similarity, a logistic curve fitting approach was used (Fig 1D). This approach allowed for the identification of drug–gene pairs where the data conformed well to the expected dose‐dependence relationship. We quantified the hit quality using the fit quality together with the maximum absolute correlation score observed at any point in the dose–response relationship (Fig 1E).

**Figure 2:**
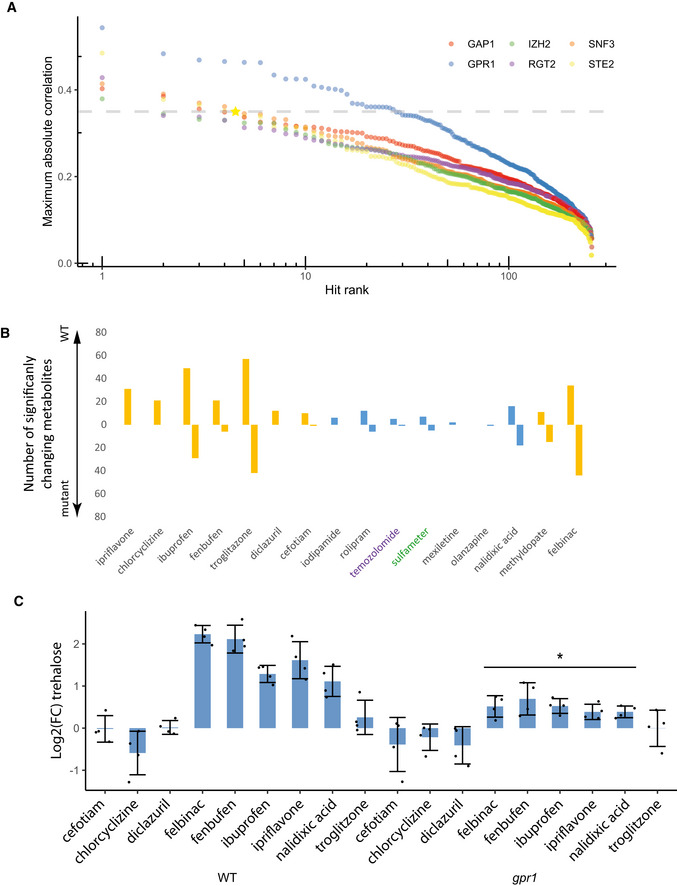
Hit characterization for drug–target predictions For drug–gene comparisons with a logistic fit quality in the top 10% of all those compared for each gene, the maximum absolute Spearman correlation coefficient for each hit comparison is shown in descending order. The horizontal dashed line indicates the maximum absolute correlation coefficient for the comparison of the metabolome of cells treated with the STE2 agonist alpha factor to STE2 overexpression conditions. The position of a STE2 to alpha factor comparison is indicated with a yellow star.The number of metabolites that change significantly when wild‐type yeast and mutants in the predicted target are treated with the indicated drugs at a concentration of 10 µM is shown. Drugs targeting GPR1 are written in black text, with drugs targeting SNF3 and RGT2 written in purple and green, respectively. Orange filled bars are predicted antagonists for GPR1.The levels of trehalose compared with a DMSO‐treated control are shown for both wild‐type yeast and *gpr1* strains treated with predicted antagonists for GPR1. The bar height indicates mean values, and error bars indicate the standard deviation of the mean. Asterisks indicate a *P*‐value of < 0.05 from a two‐sided Student’s *t*‐test (*n* = 4 biological replicates) when comparing the WT and *gpr1* responses to the drug. Results are representative of 3 independent experiments.

Drug–target pairings with logistic curve fit qualities that were in the top 10% for each gene were selected and ranked based on their maximum absolute Spearman correlation coefficients (Fig 2A). When the alpha factor peptide, an agonist for STE2, was included in these analyses it ranked in the top 1.6% of filtered comparisons for STE2 (Fig 2A) and only 0.04% of all comparisons of drugs to STE2 ranked higher than alpha factor in both fit quality and maximum absolute Spearman correlation (Appendix Fig S4A for STE2 and Appendix Fig S4B all targets). In addition, metabolites with altered abundances under alpha factor treatment could be seen to show altered levels as STE2 expression was modulated (Appendix Fig S1). This demonstrates the ability of this approach to identify known ligand–target relationships. At least one drug per gene passed the fit threshold and had a maximum absolute correlation that was greater than that of the alpha factor positive control, although top ranking GPR1 hits exhibited larger correlation coefficients than for the other genes tested (Fig 2A). Among the highest ranking hits based on maximum Spearman correlation coefficients, ~60% of the hit dose–response relationships showed a negative slope (Appendix Fig S4C), although unfiltered fits did not show the same skew (Appendix Fig S4D). This suggests that the majority of the highest ranking hits are acting in an antagonistic manner toward their target protein.

### Chemical genetic analysis of metabolome response to drug treatments

Since the hits’ effects on the metabolome in the chemical screen are predicted to be due to perturbation of their putative target, it would be expected that mutants with deleted target‐encoding genes would show an impaired response to the drug. To test whether this is the case for the drug–target predictions, both wild‐type and loss‐of‐function mutants in the putative target of the chosen drugs were treated with a subset of 15 of the highest ranking hit compounds, subject to the filters described in the methods section. Since hits for GPR1 showed significantly higher maximum absolute correlations than for other targets, the tested drugs were mostly for that target. Treatments were performed at a concentration of 10 µM for 30 min, after which intracellular metabolites were extracted and measured as described above. Comparing the number of metabolites that changed significantly in abundance across genetic backgrounds showed that the wild‐type yeast exhibited more metabolite changes than the mutants in 12 of the 15 cases (Fig 2B), consistent with the hypothesis that those drugs act on their target pathway. These results were seen for at least one drug predicted to target each of the three tested proteins. This suggests that this novel method has the promise of effectively identifying hit compounds across a range of different targets. In order to confirm that drug–gene interaction predictions were robust, more focused attention was given next to drugs predicted to target GPR1.

Focusing on the metabolome changes for the nine predicted antagonists for GPR1 that were tested, we were able to observe a significant increase in the amount of the disaccharide trehalose in many of the cases when wild‐type was treated with those antagonists (Fig 2C). This is consistent with the known effect of sugar signaling through GPR1 on trehalose (Iyer *et al*, 2008), as well as the effect of induced expression of GPR1 on disaccharide levels (Appendix Fig S1). For all of the antagonists that increased trehalose amounts, there was a reduced relative accumulation of trehalose for a *gpr1* mutant treated with the drug compared with the wild‐type case (Fig 2C). This is consistent with drugs acting through the antagonism of GPR1 signaling.

In order to confirm the results of the flow‐injection analysis that was used to generate hits, metabolome extracts were collected again for yeast treated with GPR1 antagonists and with induced receptor expression. These samples were measured using a liquid‐chromatography mass spectrometry approach. As mentioned earlier, the correlations between drug treatments and different strengths of gene overexpression were calculated for each drug and gene. When the hit drug–gene relationships were evaluated, the obtained results were very similar for all comparisons except for felbinac, which still showed a positive correlation (Appendix Fig S5). This demonstrates the robustness of the underlying measurements that were used to generate the tested predictions.

**Figure 3:**
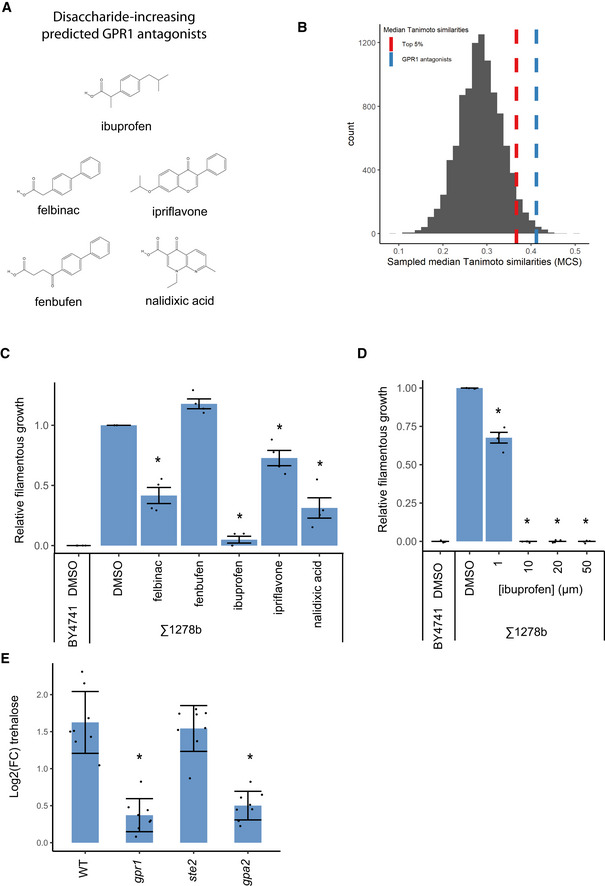
Hit validation for drug–target relationship predictions The chemical structures of predicted antagonists for GPR1 that showed a relative increase in trehalose in panel b are shown.The distribution of the median maximum common substructure Tanimoto similarity for 10,000 random selections of five compounds from the Prestwick library are shown in black. The dotted red line indicates the top 5% of values, and the dotted blue line indicates the median similarity score for the predicted antagonists for GPR1 that showed a relative increase in trehalose in (Fig 3B), which are pictured in (Fig 3C).The average quantified filamentous growth is shown for BY4741 and Σ1278b yeast spotted onto media supplemented with either a vehicle control (DMSO 0.1% (v/v) or the indicated compounds at a concentration of 20 µM across four biological replicates. Error bars represent the standard deviation of the mean between the averages of the replicates, and asterisks indicate *P*‐value < 0.05 for a two‐sided Student’s *t*‐test comparing normalized values to the DMSO control (*n* = 4 biological replicates).The average quantified filamentous growth is shown for BY4741 and Σ1278b yeast spotted onto media supplemented with either a vehicle control (DMSO 0.1% (v/v) or the indicated concentrations of ibuprofen. Error bars represent the standard deviation of the mean between the averages of the replicates, and asterisks indicate *P*‐value < 0.05 for a two‐sided Student’s *t*‐test comparing normalized values to the DMSO control (*n* = 4 biological replicates).The average relative amount of trehalose in ibuprofen‐treated cells compared with a DMSO treatment is shown. The bar height indicates mean values, error bars indicate the standard deviation of the mean, and asterisks indicate *P*‐value < 0.05 for a two‐sided Student’s *t*‐test comparing to the WT control (*n* = 8 biological replicates). Results are representative of three independent experiments.

The structures of the five predicted GPR1 antagonists that significantly increased trehalose levels include several members with similar chemical structures (Fig 3A). Since drugs with similar effects are expected to have similar structures, we decided to investigate whether the structures of putative antagonists are more similar than would be expected by chance alone. To do this, we calculated the maximum common substructure (MCS) Tanimoto score (Chen & Reynolds, 2002) for the five predicted GPR1 antagonists. In addition, over 10,000 iterations of a group of five random compounds from the Prestwick were sampled and their chemical similarities were computed. The distribution of median MCS scores (Fig 3B) showed that the median MCS score for the GPR1 antagonists is more similar than would be expected from random. Although the structural similarity is mostly seen between ibuprofen, fenbufen, and felbinac; the level of structural similarity is larger than what would be expected by chance. This provides support of the assertion that the set of compounds that are able to increase disaccharide levels may share a molecular target.

### Predicted GPR1 antagonists phenocopy known *gpr1* phenotypes

One of the specific phenotypes of yeast lacking a functional copy of GPR1 is the reduction of filamentous growth. In order to test whether the putative GPR1 antagonists were capable of phenocopying the mutant in this regard, a plate washing assay was used (Cullen, 2015). Since BY4741 is incapable of filamentous growth it was washed away with no residual growth (Appendix Fig S6). On the other hand, the DMSO‐treated Σ1278b *S. cerevisiae* strain, which is capable of filamentous growth, was able to penetrate the agar and show residual scars in the agar after washing. Mannose, a natural antagonist for GPR1 (Lemaire *et al*, 2004), was confirmed to be able to block the invasive growth of Σ1278b, demonstrating the ability of the assay to recover the effect of a known antagonist (Appendix Fig S6). Most of the Σ1278b yeast treated with 20 µM of the putative antagonists also showed reduced filamentous growth (Fig 3C), with ibuprofen showing the most striking reduction in filamentous growth. Indeed, ibuprofen was able to reduce filamentous growth with treatments concentrations as low as 1 µM (Fig 3D). This reduction in a phenotype associated with GPR1 function is consistent with the role of these drugs as novel antagonists for GPR1 signaling.

Finally, we aimed to more clearly test whether the action of ibuprofen occurred via a reduction in signaling through its downstream G‐protein, GPA2 (Kraakman *et al*, 1999). As described earlier, when *gpr1* mutants were treated with ibuprofen, the trehalose response was dampened compared with WT yeast. This was also observed for its G‐protein GPA2 (Fig 3E). In addition, a mutant in the GPCR STE2 showed a normal trehalose response to ibuprofen treatment (Fig 3E). This demonstrates that the effect of *gpr1* and *gpa2* mutations on the response of trehalose to ibuprofen is not a general property of disrupted signaling through GPCRs in the cell and provides further evidence the iburprofen acts through the GPR1‐GPA2 signaling system.

Of the 15 hit drugs that were tested, only two failed to show a positive result in at least one of the validation experiments. This shows that the prediction strategy is strongly enriching for high quality hits. Of the nine predicted GPR1 antagonists whose activities were tested, two of them showed positive results in all subsequent validation experiments (ibuprofen and ipriflavone). This represents a minimum recall rate of 22% for validating newly predicted antagonists of GPR1 based solely on the comparison of drug and mutant metabolome profiles.

### Prediction of drug target relationships for non‐membrane proteins

In order to test the broader applicability of this approach, as well as its suitability to genome‐scale deployment, a collection of 86 genes were selected from the non‐essential Yeast Estradiol strains with Titratable Induction (Arita *et al*, 2021) (YETI) library. This collection of genes was composed primarily of enzymes as well as receptor‐related proteins due to their druggable nature and their importance as pharmacological targets. In addition, this gene‐list was chosen such that it was comprised mostly of non‐membrane proteins. This was done in order to test the generalizability of the approach outlined earlier beyond membrane proteins.

**Figure 4:**
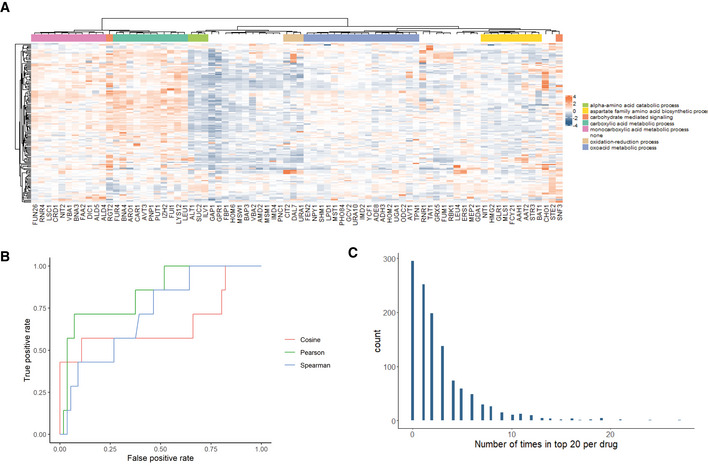
Drug–target predictions for non‐membrane proteins Average z‐scored metabolome profiles are clustered for all estradiol‐induced overexpression strains. GO enrichment analysis is performed on all clusters with the most widely shared biological process that is significantly enriched within that cluster indicated.ROC curves indicate the recovery of 7 true‐positive drug–target interactions within the dataset compared with the number of false discoveries from the remaining 1280 drugs within the dataset. The recovery of expected interactions is shown using Spearman correlation, Pearson correlation, and cosine similarity. These similarity metrics allow for an area under the curve of 0.72, 0.84, and 0.66 for those similarity metrics, respectively.The number of occurrences where each drug within the library is in the top 20 ranking interactions for each target is tabulated for drugs that cause significant (*P*‐values < 0.05) changes in at least 5 metabolites.

The overexpression yeast strains were grown to an OD_600_ of ~0.7, at which time they were treated with 10 µM of β‐estradiol or the vehicle control DMSO. Cultures were treated with estradiol for 1.5 h prior to extraction, at which point metabolites were extracted as described earlier. Similarly, metabolomics data were generated by flow‐injection mass spectrometry as described earlier. Upon z‐scoring, these metabolome profiles were subjected to hierarchical clustering to assess the recovery of expected relationships. In order to take an unbiased approach, GO biological process enrichment was performed on each cluster in order to determine whether mutants whose metabolome profiles cluster together have related functions. GO term enrichment was seen for almost all clusters in the analysis (Fig 4A). These clusters showed a greater GO enrichment than would be expected by chance, with empirical *P*‐values of < 0.001 as determined through 10,000 rounds of data shuffling and GO enrichment. These data demonstrate that that the inducible overexpression generates metabolome profiles that capture the biological roles of the genes that are overexpressed.

The genes selected for these experiments included seven cases for which known drug–target relationships are established (Dataset EV8), allowing for a systematic analysis of the recovery of true‐positives within the dataset. The overexpression metabolome profiles were compared with those of yeast treated with the compounds from the Prestwick library, as well as those treated with their known agonists or antagonists. Because yeast were treated with only one concentration of estradiol, the logistic‐fitting approach described above was not applicable. Instead, the similarity of each z‐scored average metabolome profile was simply compared with each drug treatment in the induced or uninduced state. For antagonists, a positive similarity was expected between the genetic treatment and the drug treatment, while an agonist would be expected to have a negative similarity. In the case of induction, agonists were expected to have a positive similarity to the estradiol treatment and antagonists would rather be expected to have a negative similarity.

Pearson correlation, Spearman correlation, and cosine similarity were evaluated for their ability to recover positive control interactions within the dataset. The similarity scores of each drug within the positive control and Prestwick dataset were ranked per mutant, and the ranking of the positive control compounds in their comparison was used in order to produce a receiver operating characteristic (ROC) curve for each set of comparisons. Strikingly, when any of the three similarity metrics were used to test the recall of the positive controls with the estradiol‐treated yeast, the interactions were found to rate remarkably highly within the dataset. This resulted in an area under the curve with respect to recovery of true positive versus false positives of between 0.74 for Spearman similarity, 0.66 for cosine similarity, and 0.84 for Pearson correlation (Fig 4B). This may in part be due to strong changes in relatively few metabolites underlying some positive control interactions (Appendix Fig S7). By comparison, positive controls were recalled significantly more poorly for the comparisons with the uninduced yeast (Appendix Fig S8, AUC 0.63). This suggests that the employment of inducible overexpression more effectively captures the relationship between drugs and their targets, at least under the relatively short drug treatments that were employed in this study. In addition, when the analysis was performed either with looser restrictions for which ions to include in the analysis, the recovery of positive controls was not improved (maximum AUC 0.8, Appendix Fig S9).

Akin to the dose‐dependent analysis performed earlier, this analysis captured known true‐positive drug–target interactions. Next, we wanted to determine whether the drug–target predictions that were obtained across the dataset were relatively homogenous or whether different inducible overexpression strains showed specific patterns of drugs that are predicted to target them. In order to assess this, we determined the number of times that each given drug was found to be in the top 20 drugs for any of the mutants in the analysis (Fig 4C). This distribution demonstrated that almost 300 of the drugs were not in the top 20 for any gene, but the second most common number of top 20 appearances per drug was 1. This suggests that this approach can generate diverse drug–target prediction for a range of different target types.

As discussed above, there is a general expectation that small molecules that are able to modify the activity of a particular target are more likely to share structural features than drugs which do not. In order to test whether our top predictions showed a pattern of structural similarity which is greater than would be expected by chance, we determined the median structural similarity for top ranking hits for each gene, and compared that distribution of values to those which arise through chance alone. Top hits showed a median structural similarity that was significantly greater than that observed in randomized data (Appendix Fig S10), suggesting that our pipeline not only recaptures known drug–target interactions but also recovers expected relationships between structurally similar drugs.

## Discussion

In this work, we present a conceptually novel method for systematic prediction of drug–target interactions that is based on the similarity of metabolome profiles in inducible overexpression mutants and drug‐treated cells. Through this approach we demonstrated for the first time that high‐throughput metabolomics can recover drug–target interactions in a eukaryotic cell and that this is possible for cell surface signaling proteins as well as cytosolic proteins. This approach has a number of advantages for identification of hit compounds in eukaryotes. Unlike traditional chemical screens, it has the potential to generate robust, whole‐genome predictions of drug–target interactions. Despite this scalability, our approach also shows a true‐positive rate that is in line with more classical screening approaches. In all, 80% of the predicted drug–target interactions that were tested through chemical genetics gave a positive result. This demonstrates a high recall rate of compounds from a screening phase to their first validation. With further analysis of the effects of drugs acting as antagonists for GPR1, we found that 22% of hit drugs gave positive results of activity on all three assays that were performed. Similar rates of attrition for hit compounds can be found in traditional chemical screens when hits are analyzed for activity using approaches that are orthogonal to the initial screen (Swingle *et al*, 2017). Furthermore, when we broadened our approach we were able to recover true‐positive drug–target interactions with an AUC of 0.84, demonstrating the ability of the approach to recover previously known drug–target relationships. Because our approach uses an untargeted methodology, more of the 13,000 ions that were detected can eventually be annotated to metabolites when better databases become available. In addition, the data presented in this article serve as a resource in which the effects of 1,280 drugs on the yeast metabolome are made available for future drug–target relationship predictions or for other purposes. Importantly, we demonstrated the effectiveness of our approach for membrane proteins that are intrinsically difficult to work with from a biochemical perspective (Carpenter *et al*, 2008). This is a tremendous problem from the perspective of chemical screening, since membrane proteins are important for understanding cell signaling systems and also are important drug targets (Rask‐Andersen *et al*, 2014) and play key roles in drug toxicity for proteins such as in solute carrier proteins (Girardi *et al*, 2020). Because our approach does not require biochemical manipulations, it avoids this pitfall.

We developed an *in vivo* screening approach, and thus it is possible that the drugs are metabolized by the cell and that a metabolized form is interacting with the target. However, this can be an advantage for *in vivo* screening approaches. For example, leads from *in vitro* screening approaches can prove ineffective *in vivo* due to metabolism of the drug. Further, when targeting intracellular proteins it is desirable to use a screening approach such as ours that relies on the ability of small molecules to enter the cell since this is a key aspect of the efficacy of the drug *in vivo*. The increasingly granular connections made between mechanisms of uptake and drug sensitivity highlight the importance of not taking this for granted (Girardi *et al*, 2020). These strengths highlight the efficacy of this method for identifying drug–target interactions.

The chemical library chosen for this study is composed of compounds with known mechanistic targets in various organisms. However, the five identified antagonists for GPR1 have no previously known targets in yeast. Ipriflavone is a synthetic isoflavone, and nalidixic acid is a quinolone antibiotic. Most intriguingly, the last three of the drugs (ibuprofen, fenbufen, and felbinac) are non‐steroidal anti‐inflammatory drugs (NSAID) targeting cyclooxygenase in higher cells. Previous reports have indicated that ibuprofen can reduce the formation of biofilms by the pathogenic fungus *Candida albicans* (Alem & Douglas, 2004), and that combinatorial treatment of the yeast with ibuprofen and fluconazole can prove more effective than fluconazole treatment alone (Costa‐de‐Oliveira *et al*, 2015). Here, we demonstrated that ibuprofen can reduce filamentous growth, and that it antagonizes GPR1. Filamentous growth in *S. cerevisiae* has been used to understand the mechanisms of biofilm formation in pathogenic yeasts (Cullen & Sprague, 2012), and since the *C. albicans* genome encodes a GPR1 homolog, these results suggest that ibuprofen may disrupt biofilm formation in *C. albicans* through affecting the activity of GPR1. These results suggest the potential utility of developing analogs of ibuprofen that could serve as higher potency antagonists to specifically inhibit *C. albicans* GPR1 and thus reduce the burden of this pathogen.

This approach is not free of limitations. Although it could be used to identify agonists, it identified antagonists at a higher rate. This is a common result for chemical screens (Hughes *et al*, 2011) and may be caused by partial agonists acting as a competitive antagonists since an endogenous ligand is present in the media for most of the targets (Detry *et al*, 1984). Another potential limitation is that many more high ranking hits were discovered for GPR1 than for the other targets in the study. GPR1 showed strong overexpression inducer dose‐dependent changes in metabolism, which allowed for strong matching between the metabolomes of drug treatments with those of overexpression conditions for GPR1 compared with the other genes in the panel. This is expected, since the matching of drugs and genes depends on the quality of the metabolome response to the genetic perturbation. This is, however, unlikely to be a significant limitation because previous results from other organisms have shown that most loss‐of‐function mutations have a measureable effect on the metabolome (Fuhrer *et al*, 2017). In addition, this approach makes use of both overexpression and loss‐of‐function conditions, meaning that it can still work in circumstances where redundancy hides the influence of a loss‐of‐function mutation on the metabolome. Another potential limitation lies in the selection of media conditions. Drug treatments or mutations that might have a strong consequence for the metabolome can be hidden in growth conditions where a particular metabolic pathway is not being used. Thus it is important to select conditions such that they maximize the chance of being able to detect metabolome responses for key classes of targets, but this consideration is equally relevant for other *in vivo* screening approaches. Finally, as with all chemical screening approaches, the predicted drug–target relationships are not responsible for the entirety of the effect of any given small molecule on the cell. Indeed, the small molecules from the library would be expected to affect a collection of targets (Mestres *et al*, 2009). However, we demonstrated that the response of the cell to small molecule treatments was at least in part contingent of the presence of its putative target thus demonstrating that the effect of the drugs are generally mediated at least in part through that target. This makes the approach a sensible way of identifying lead compounds for chemical probe or drug development.

Taken together, these results raise the possibility of predicting drug–target interactions on a genome‐wide scale and thus offers a roadmap to a broad catalogue of predicted drug–target relationships across the genome. Such a catalogue would be invaluable for speeding the rate of discovery of chemical probes that can be used to tease apart signaling systems by selectively and dynamically altering the activity of targets within the cell, but would also speed the rate of lead compound identification for drug development.

## Materials and Methods

### Reagents and Tools table

**Table:** 

Reagent/resource	Reference or source	Identifier or catalog number
**Experimental models**		
Yeast artificial transcription factor overexpression system	(McIsaac *et al*, 2013)	N/A
YETI non‐essential library collection	(Arita *et al*, 2021)	N/A
Prestwick Chemical Library: 1,280 drug collection	Prestwick Chemical	N/A
**Chemicals, enzymes, and other reagents**		
Yeast extract	BD Biosciences	288630
Bacto‐peptone	BD Biosciences	214530
G 418 disulfate salt	Sigma‐Aldrich	A1720
Ammonium sulfate	Sigma‐Aldrich	A4418
Yeast nitrogen base	BD Biosciences	233530
d‐(+)‐glucose	Sigma‐Aldrich	G8270
Dextrose	Sigma‐Aldrich	D9434
Agar	BD Biosciences	214530
Amino acid dropout supplement without uracil	Sigma‐Aldrich	Y1501
β‐Estradiol	Sigma‐Aldrich	E8875
Dimethylsulfoxide (DMSO)	Sigma‐Aldrich	276855
Ibuprofen	Sigma‐Aldrich	I4883
α1‐Mating factor	Sigma‐Aldrich	T6901
Thaumatin	Sigma‐Aldrich	T7638
Atorvastatin	Sigma‐Aldrich	1044516
Hydroxyuracil	Sigma‐Aldrich	H8627
Aminooxyacetate	Sigma‐Aldrich	C13408
Pentostatin	Sigma‐Aldrich	SML0508
Fluvastatin	Sigma‐Aldrich	PHR1620
HPLC‐grade acetonitrile	Sigma‐Aldrich	34998
HPLC‐grade methanol	Sigma‐Aldrich	34885
HPLC‐grade water	Sigma‐Aldrich	1153331000
Trehalose quantification kit	Megazyme	K‐TREH
**Software**		
Matlab R2018b	The MathWorks, Inc.	N/A
Python 3.9.5	https://www.python.org/	N/A
Spyder 4.1.4	https://www.spyder‐ide.org/	N/A
SciPy 1.5.0	(Virtanen *et al*, 2020)	N/A
Scikit learn 0.23.1	https://scikit‐learn.org/	
R 4.0.3	https://cran.r‐project.org/	N/A
RStudio 1.2.1335	https://www.rstudio.com/	N/A
Clusterprofiler 3.16.1	(Yu *et al*, 2012; Wu *et al*, 2021)	N/A
coop 0.6‐3	https://github.com/wrathematics/coop	N/A
ImageJ	(Carpenter, 2007; Rueden *et al*, 2017)	N/A
**Other**		
6550 iFunnel Q‐TOF mass spectrometer	Agilent	N/A
MPS2 autosampler	Gerstel	N/A
1290 Infinity LC System	Agilent	N/A
InfinityLab Poroshell 120 HILIC‐Z column (2.1 × 100 mm, 2.7 µm)	Agilent	679775‐924
InfinityLab Poroshell 120 HILIC‐Z guard column (2.1 mm, 2.7 µm)	Agilent	821725‐947
Sunrise plate reader	Tecan	N/A

### Methods and Protocols

#### Yeast cultivation

Yeast cultivated in liquid culture were grown at a temperature to 30°C shaking at a frequency of 250 rpm. For solid plate growth, yeast were allowed to grow at 30°C. Cloning was performed using YPD plates with G‐418 (10 g/l yeast extract (BD Biosciences: 288630), 20 g/l Bacto‐peptone (BD Biosciences: 211830), 5 g/l agar (BD Biosciences: 214530), 300 µg/ml G‐418 (Sigma‐Aldrich: A1720)). Yeast grown for Figs 1, 2, 3 were cultivated in SD media (5 g/l ammonium sulfate (Sigma‐Aldrich: A4418), 1.7 g/l Yeast Nitrogen base (BD Biosciences: 233530), 20 g/l D‐(+)‐glucose (Sigma‐Aldrich: G8270)) with uracil amino acid dropout (1.94 g/l Yeast Synthetic Drop‐out Medium Supplements without uracil (Sigma‐Aldrich: Y1501)). In Fig 4A and B, growth was in SD media (see above) with histidine dropout [1.94 g/l Yeast Synthetic Drop‐out Medium Supplements without histidine (Sigma‐Aldrich: Y1751)].

#### Strains used for study

All β‐estradiol inducible strains constructed for this study were generated using the system described by McIsaac (McIsaac *et al*, 2013). Briefly, the KanMX resistance gene as well as the synthetic β‐estradiol responsive Z4EV promoter was amplified from the pMN10 plasmid. This was done with primers with 5′ ends matching the sequence 300 base pairs upstream of the target gene’s transcriptional start site for the forward primer and matching the reverse complement of the start of the open reading from for the reverse primer. The generated PCR products were transformed into the DBY12416 strain using the LiAC/SS carrier DNA/PEG method (Gietz & Schiestl, 2007a). Transformants were selected through growth on YPD + G‐418 plates and correct replacement of the native promoter was confirmed by PCR and DNA sequencing. The wild‐type control strain for overexpression strain was constructed in the same way, but only the KanMX gene was amplified and it was inserted in an intergenic region upstream of the HO locus. For chemical‐genetic experiments described in Figs 2B and C, and 3E, deletion mutants in a haploid BY4741 background were used. For those experiments, the strains were transformed with the pHLUM minichromosome (Mülleder *et al*, 2012) to restore prototrophy using the transformation approach described above. In this case, selection was performed on YNB plates supplemented with ammonium sulfate and glucose.

#### Metabolite extraction

For all metabolite extractions, cultures were grown to a target OD_600_ of 1.0 with at least two cell doublings between inoculation and harvesting. Cultivation was performed in deep 96‐well plates at a volume of 1.2 ml. OD_600_ was monitored 4 times, leading to a final volume of 1 ml at the time of sampling. For overexpression experiments, the yeast were treated with the indicated concentrations of β‐estradiol dissolved in DMSO (Sigma‐Aldrich: E8875, final concentration 0.1%) for 1.5 and 3 h prior to sampling. Extractions were performed at an OD_600_ of ~1.0. For dose–response‐induced overexpression experiments, biological replicates were the result of separate wells within the same plate that were inoculated from independent precultures. For the overexpression experiments outlined in Fig 4, biological replicates represent cultures inoculated and cultivated in separate 96‐well plates. For drug treatments, the yeast were treated with the drugs at a concentration of 10 µM, or 100 µg/ml for alpha factor, for half an hour prior to sampling. The final concentration of the vehicle (DMSO) in these cases was also 0.1% (v/v). Within drug‐treatment overexpression experiments biological replicates represent cultures inoculated and cultivated within separate 96‐well plates. Treatment concentrations and pairings for positive control drug treatments are reported in Dataset EV8. At the time of harvesting the samples were centrifuged for 1 min with a force of 2,254 *g*. The supernatant was discarded and 150 µl of cold extraction solution [40% (v/v) HPLC‐grade acetonitrile (Sigma‐Aldrich: 34998), 40% (v/v) HPLC‐grade methanol (Sigma‐Aldrich: 34885), 20% (v/v) HPLC‐grade water (Sigma‐Aldrich: 1153331000)] was added to the residual cell pellet. The extraction was covered and placed at −20°C for 1 h. After 1 h, the deep‐well plates holding the extraction were centrifuged for 1 min at 2,254 rcf before 100 µl of the supernatant was taken and transferred into conical 96‐well plates (Huber lab: 7.1058) before being sealed (Huber lab: 7.0745) for long‐term storage at −80°C until the time of measurement.

#### Flow injection time‐of‐flight mass spectrometry

Mass spectrometry was performed using an Agilent 6550 Series quadrupole time‐of‐flight mass spectrometer (Agilent) by and adaptation of the method described by Fuhrer *et al* (2011) Analysis was performed using an Agilent 1100 Series HPLC system (Agilent) was coupled to a Gerstel MPS 3 autosampler (Gerstel). The mobile phase flow rate was set of 0.15 ml/min, with the isocratic phase composed of 60:40 (v/v) isopropanol and water buffered to a pH of 9 with 4 mM ammonium fluoride. Online mass axis correction was performed with taurocholic acid and Hexakis (1H, 1H, 3H‐tetrafluoropropoxy)–phosphazne) within the mobile phase. The instrument was run in 4 GHz mode for maximum resolution while collecting mass spectra between 50 and 1,000 *m/z*.

#### Analysis of flow injection mass spectrometry data

Mass spectrum centroiding, merging, and ion annotation was performed as described in Fuhrer *et al* Raw ion intensities can be found in Dataset EV1. (Fuhrer *et al*, 2011) Data normalization and analysis was performed in Python using the Pandas package (McKinney, 2019). Briefly, the datasets were filtered for outliers in terms of OD_600_ at the time of sampling as well as in total ion current. Previously measured mass spectra for the compounds in the Prestwick chemical library were used to filter out drugs that were misannotated as metabolites. Raw ion intensities were normalized to counteract temporal drifts, as well as OD_600_ effects. To remove temporal drifts, a LOWESS regression was fitted for each ion with respect to injection sequence per measurement batch. The trend with respect to injection sequence was then subtracted, and differences in median ion intensity across the batches were similarly subtracted. OD600 normalization was accomplished through a linear regression for each ion with respect to the OD600 at the time of sampling, and removing those residual trends. Normalized ion intensities were z‐score transformed. For chemical screening samples, the z‐scores were calculated for each ion with x representing the value for the sample in question for that ion. Sigma was the standard deviation of the intensity of that ion for all samples derived from the same 96‐well plate. Mu was the average of the intensity of that ion for all samples derived from the same 96‐well plate. For dose–response overexpression samples, the *z*‐scores were calculated for each ion with x representing the value for the sample in question for that ion. Average *z*‐score were calculated for every drug and overexpression condition. Sigma was the standard deviation of the intensity of that ion for the paired estradiol‐treated WT samples. Mu was the average of the intensity of that ion for the paired estradiol‐treated WT samples. Average *z*‐scored ion intensities between the drug treatment and overexpression data sets were merged into one data set and the Spearman correlation coefficient was calculated for each drug with each overexpression condition. For the single estradiol concentration overexpression conditions described in Fig 4, z‐scores were calculated using the same approach as described for the drug‐treated cases. The effect of normalization on the variance explained by the data’s principal components is depicted in Appendix Fig S11. *z*‐Score values with ion annotation can be found in Dataset EV2. For the metabolomics data depicted in Fig 4, raw ion intensities can be found in Dataset EV7 and average z‐scored ion intensities can be found in Dataset EV9.

#### Logistic fitting of Spearman R values for hit prioritization

Logistic curve fitting was done using the optimize.curve_fit function from SciPy (Virtanen *et al*, 2020) For each overexpression and time point dose response, the data were scaled between 0 and 1 using the scikit‐learn MinMaxScaler function. A logistic curve function was then fit to the relationship between estradiol dose and scaled Spearman R values. Fits were done using a 2‐point method for computing the Jacobian matrix. A starting point of 2 for the inflection point and steepness of 0 was chosen. The returned optimized values as well as the estimated covariance were stored, with the covariance metric being used to rank fits on their quality. Logistic fit output values can be found in Dataset EV3. Comparisons with a covariance score indicating a fit in the top 10% of comparisons were considered as potential hits. As described above, this level is sufficient to recover the true positive interaction of alpha factor with STE2, and allows for the recovery of fit qualities of similar or marginally poorer quality. Candidates hits were further filtered based on whether they had a maximum absolute Spearman correlation as high or higher than that of the comparison of alpha factor with STE2. For further analysis, control and steroid‐related hits were discarded as artifacts of the β‐estradiol induced overexpression system. Cosine similarity was calculated using the R “coop” package, and the similarity output was used in the same analytical pipeline outlined above. Fits with an inflection point outside of the bounds of the concentrations of estradiol that were added were also discarded.

#### Liquid‐chromatography mass spectrometry

Metabolite extracts were prepared as described above. Metabolite extracts were subjected to chromatographic separation through normal phase chromatography. Separation was performed using Agilent Infinity 1290 UHPLC stack with Agilent 1100 Series binary pump with an InfinityLab Poroshell 120 HILIC‐Z column (2.1 × 100 mm, 2.7 µm, Agilent) including an InfinityLab Poroshell 120 HILIC‐Z UHPLC guard column (2.1 mm, 2.7 µm, Agilent). Samples were analyzed using an Agilent 6550 Series quadrupole time‐of‐flight mass spectrometer running in negative extended dynamic range mode. Mobile phases were 10 mM ammonium acetate pH 9 in water with 5 µM medronic acid, and 90:10 acetonitrile:water with 10 mM ammonium acetate pH 9. A flow rate of 600 µl/min was used with a total measurement duration of 5 min. Mobile phase compositions were set as described in Dataset EV4. Online mass‐axis correction was performed using purine and hexakis. Data analysis was performed in Agilent MassHunter Quantitative Analysis (Version B.07.00) with peaks chosen based on retention time matching to compounds in standard solution. 20 parts per million *m/z* windows were used for peak selection, with integration performed using spectal summation of specified time and mass windows. Average Log2 transformed fold changes were calculated between samples, and the Pearson correlation coefficient was calculated for each pairing of induced receptor overexpression and drug treatment. Peak areas for overexpression and drug treatments are reported in Datasets EV5 and EV6 respectively.

#### Structural similarity comparison

The R package ChemmineR (Cao *et al*, 2008) was used for all cheminformatics described here. SDF files were generated from the SMILES structural descriptions accompanying the Prestwick library. The pairwise MCS Tanimoto similarity score was then calculated between all the SDF representations of the drugs using a MCS algorithm, and fingerprint similarity was calculated with a pairwise Tanimoto comparison of binary fingerprints.

#### Trehalose assays

Trehalose extractions were performed as described above for metabolite extractions with the following modifications: Strains were cultivated in synthetic defined media including all amino acids, samples were washed twice with distilled water prior to metabolite extraction, and metabolite extractions were allowed to proceed for 18 h at −20°C. Trehalose measurement was performed using an enzymatic detection kit (Megazyme: K‐TREH). 20 µl of metabolite extracts were subjected to analysis, the difference in NADPH absorbance was measured with a Tecan Infinite M Nano+ plate reader before and after the addition of the trehalase enzyme to the assay in order to estimate relative residual treahalose between samples.

#### Filamentous growth assays

Filamentous growth was assayed using a plate washing assay (Gietz & Schiestl, 2007b). Briefly, 5 ml of melted YEPD agar medium (10 g/l yeast extract (BD Biosciences: 288630), 20 g/l Bacto‐peptone (BD Biosciences: 211830), 5 g/l Agar (BD Biosciences: 214530), 20 g/l Dextrose (Sigma‐Aldrich: D9434)) with a temperature of approximately 55°C was mixed with 5 µl of the indicated drugs or vehicle control in order to reach a final concentration of 20 µM or the concentrations indicated in the figure caption. The drug‐media mixture was deposited into the wells within 6 well plates and allowed to cool. For mannose experiments, 100 µl of 200 g/l D‐(+)‐mannose or a water control was added to each well prior to media addition. Both BY4741 and Σ1278b yeast were grown to stationary phase in precultures, and 5 µl of the yeast were spotted onto the solid media within the plates. The yeast were allowed to grow on the media for 3 days. The colonies were then photographed, and the surface yeast was washed away. The residual scars left in the media from the filamentous growth were then photographed (Appendix Fig S12 for images). For ibuprofen dose–response experiments, the experiments were performed at a 1 ml scale in 24‐wll plates with the residual scars photographed under transillumination (Appendix Fig S13 for images). The amount of scarring of the agar was quantified using the gel analysis tool in ImageJ (Rueden *et al*, 2017) with subtraction from an untreated well to establish baseline conditions.

#### GO enrichment analysis

YETI library yeast strains were clustered by Ward’s method based on Manhattan distance. The 86 genes in the dataset were split into 15 clusters based on the distance between the clusters and the genes within those groups were subjected to GO enrichment analysis. GO term enrichment was tested using the ClusterProfiler R package (Yu *et al*, 2012; Wu *et al*, 2021). The ratio between the occurrence of the most widespread but significantly enriched biological process term in each cluster and the background rate in the genome was calculated and the average enrichment factor for all mutants was determined. Enrichment factors were determined for both the genuine data and data where the group membership had been shuffled. When this randomization was performed 1,000 times, the empirical *P*‐value for the relative enrichment rate for the true data was found to be < 0.001.

## Author contributions

**Duncan Holbrook‐Smith:** Conceptualization; Data curation; Formal analysis; Funding acquisition; Investigation; Visualization; Methodology; Writing—original draft; Writing—review & editing. **Stephan Durot:** Investigation. **Uwe Sauer:** Conceptualization; Supervision; Funding acquisition; Writing—original draft; Project administration; Writing—review & editing.

In addition to the CRediT author contributions listed above, the contributions in detail are:

SD Cloned overexpression lines, and collected overexpression metabolomics data used in Fig 1. DH‐S Cloned overexpression lines, and collected all the experimental data excluding the contributions of SD. DH‐S analyzed all the data in the study. DH‐S and US conceived of the project. DH‐S and US wrote the manuscript with input from SD.

## Supporting information



Appendix

Dataset EV1

Dataset EV2

Dataset EV3

Dataset EV4

Dataset EV5

Dataset EV6

Dataset EV7

Dataset EV8

Dataset EV9

## Data Availability

The raw flow‐injection mass spectrometry data are deposited in the MassIVE database (https://massive.ucsd.edu) and can be accessed using the code MSV000086451. LC‐MS drug treatment raw data are available using the MassIVE code MSV000087420. LC‐MS gene overexpression data are available using MassIVE code MSV000087421. Raw mass spectrometry data for drug treatment and overexpression experiments introduced in Fig 4 are available with MassIVE codes MSV000088124 and MSV000088125 for drug treatment and overexpression data, respectively. All other data, including processed mass spectrometry data, are provided as EV Datasets, as indicated subsequently: Dataset EV1: raw ion intensities, sample metadata, and ion information for Prestwick and titrated overexpression experiments. Dataset EV2: top ion annotations and average z‐scores for Prestwick and titrated overexpression screens experiments. Dataset EV3: logistic fit output for drug and overexpression comparison. Dataset EV4: mobile phase compositions and flow‐rates for LC‐MS analysis. Dataset EV5: peak areas for LC‐MS analysis of overexpression mutants. Dataset EV6: peak areas for LC‐MS analysis of drug treatments. Dataset EV7: annotated ion intensities and sample information for mass spectrometry in Fig 4. Dataset EV8: true‐positive drug–target relationships shown in Fig 4. Dataset EV9: average z‐scored ion intensities for mass spectrometry shown in Fig 4.
