# Supplementary material for: High‐throughput metabolomics predicts drug–target relationships for eukaryotic proteins
Source: Mol Syst Biol. 2022 Feb 23;18(2):e10767. doi: 10.15252/msb.202110767 (PMC8864444; doi:10.15252/msb.202110767)
Supplement: Supplementary file 1 — Appendix [file MSB-18-e10767-s011.pdf]

Appendix

High-throughput metabolomics predicts drug-target relationships for eukaryotic proteins

Duncan Holbrook-Smith, Stephan Durot, Uwe Sauer

Table of Contents

Item	Title	Page
Appendix Figure S1	Dose-dependent patterns of metabolite abundance are seen across receptor overexpression conditions	2
Appendix Figure S2	The effect of a collection of 12 drugs from the Prestwick library on the indicated metrics at 5 time points is shown	3
Appendix Figure S3	Effect of indicated drugs on metabolite abundances	4
Appendix Figure S4	The distribution of coefficients resulting from the fitting of drug-gene similarity	5
Appendix Figure S5	LC-MS analysis recaptures drug-gene relationships for working GPR1 antagonists	6
Appendix Figure S6	The effect of the known GPR1 antagonist mannose on filamentous growth	7
Appendix Figure S7	Bivariate scatterplots of drug-gene true positive pairs	8
Appendix Figure S8	The recovery of true-positive interactions for uninduced YETI strains	9
Appendix Figure S9	Recovery of true positives with altered ion inclusion criteria	10
Appendix Figure S10	Drugs predicted to share same target share structural similarity	11
Appendix Figure S11	Effect of predicted GPR1 antagonists on filamentous growth	12
Appendix Figure S12	Dose-response of filamentous growth with ibuprofen treatment	13
Appendix Figure S13	Effect of normalization of dataset variance	14

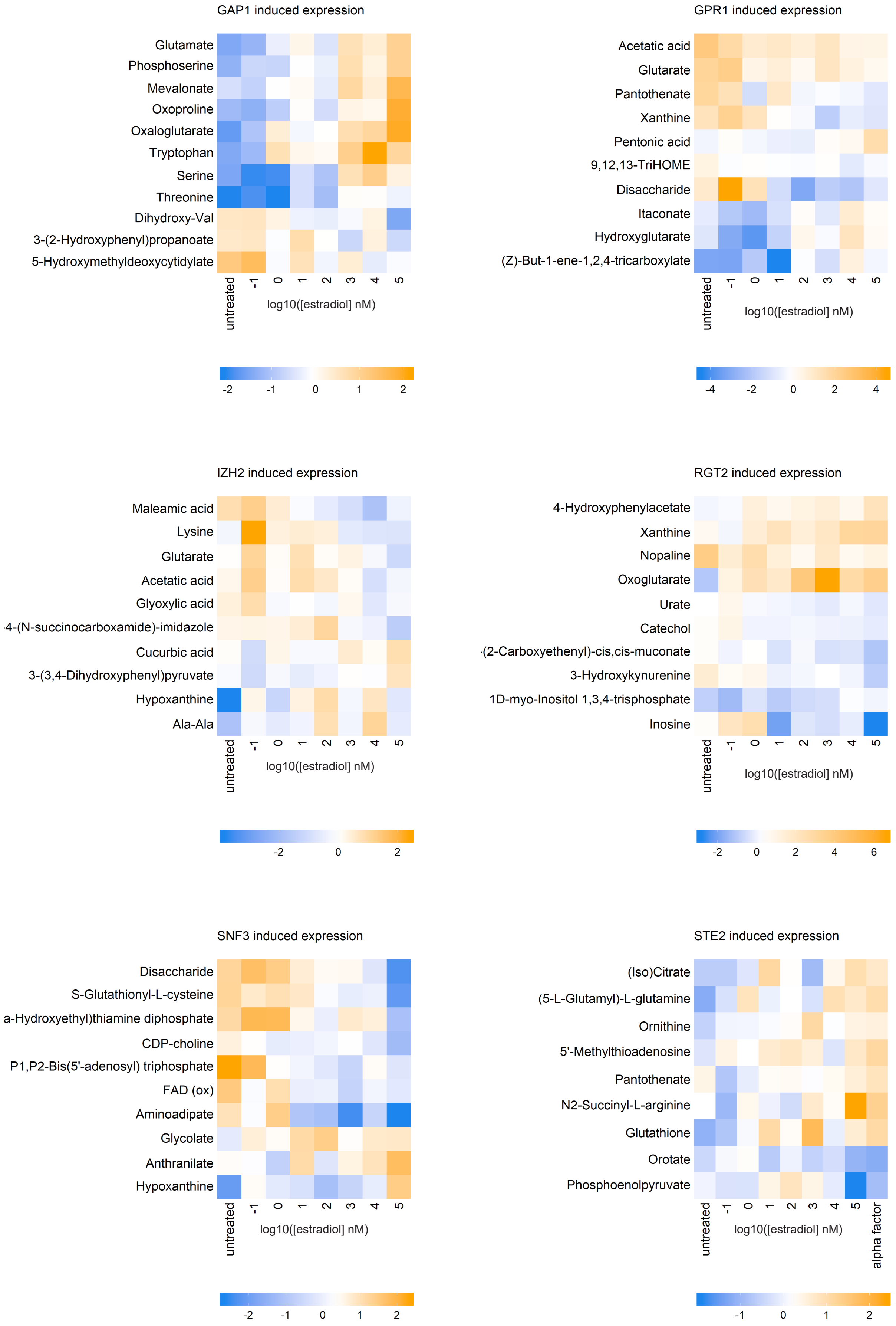

Appendix Figure S1: Dose-dependent patterns of metabolite abundance are seen across receptor overexpression conditions

The effect of increasing concentrations of the expression inducer ß-estradiol on the z-scored abundance of the indicated annotated metabolites after 1.5 hours is shown. Z-scores are calculated relative to the matched ß-estradiol treatment of the reference strain lacking the synthetic overexpression promoter. For STE2, the effect of treatment with 10 µg/mL alpha factor in a separate experiment is also shown for comparison.

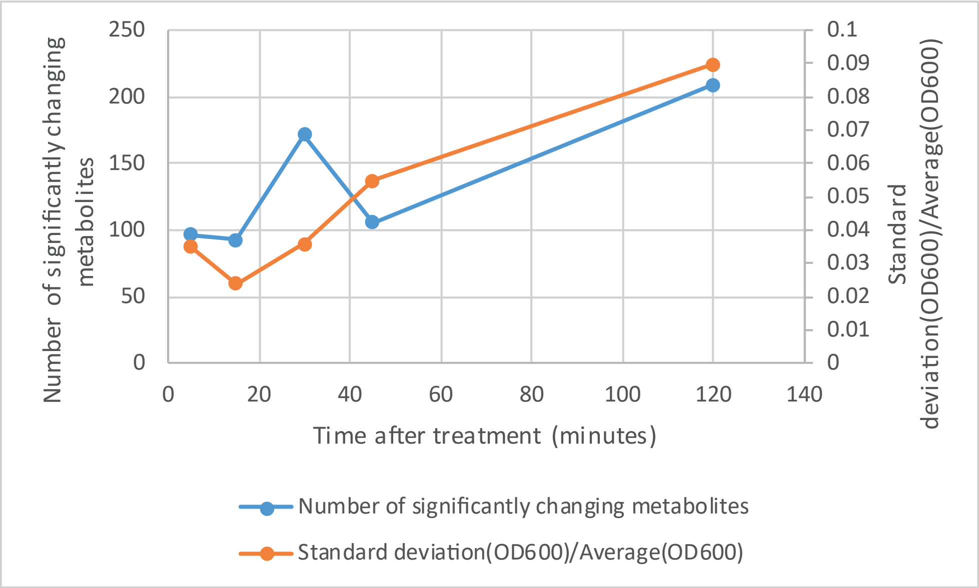

Appendix Figure S2: The effect of a collection of 12 drugs from the Prestwick library on the indicated metrics at 5 time points is shown

Signficantly changing metabolites are based on a Log2(FC) compared to an untreated control with a p-value < 0.05. OD_{600} measurements were made immediately prior to sampling.

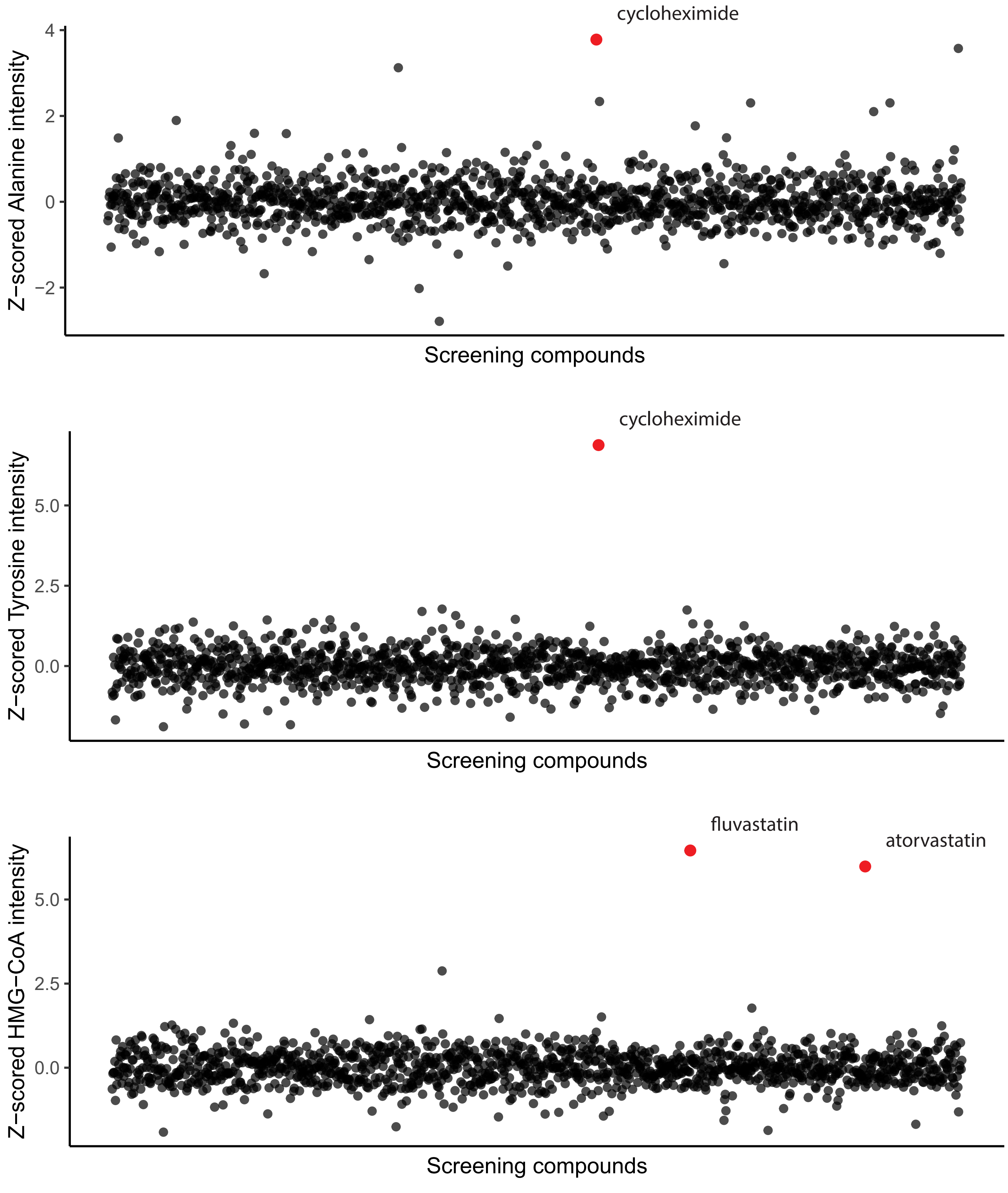

Appendix Figure S3: Effect of indicated drugs on metabolite abundances
The relative average relative amount of the indicated metabolites under treatment with all 1280 drugs from the Prestwick chemical library is shown. Z-scores were calculated relative to the screening plate. Drugs with known effects on the abundance of the indicated metabolites are highlighted in red.

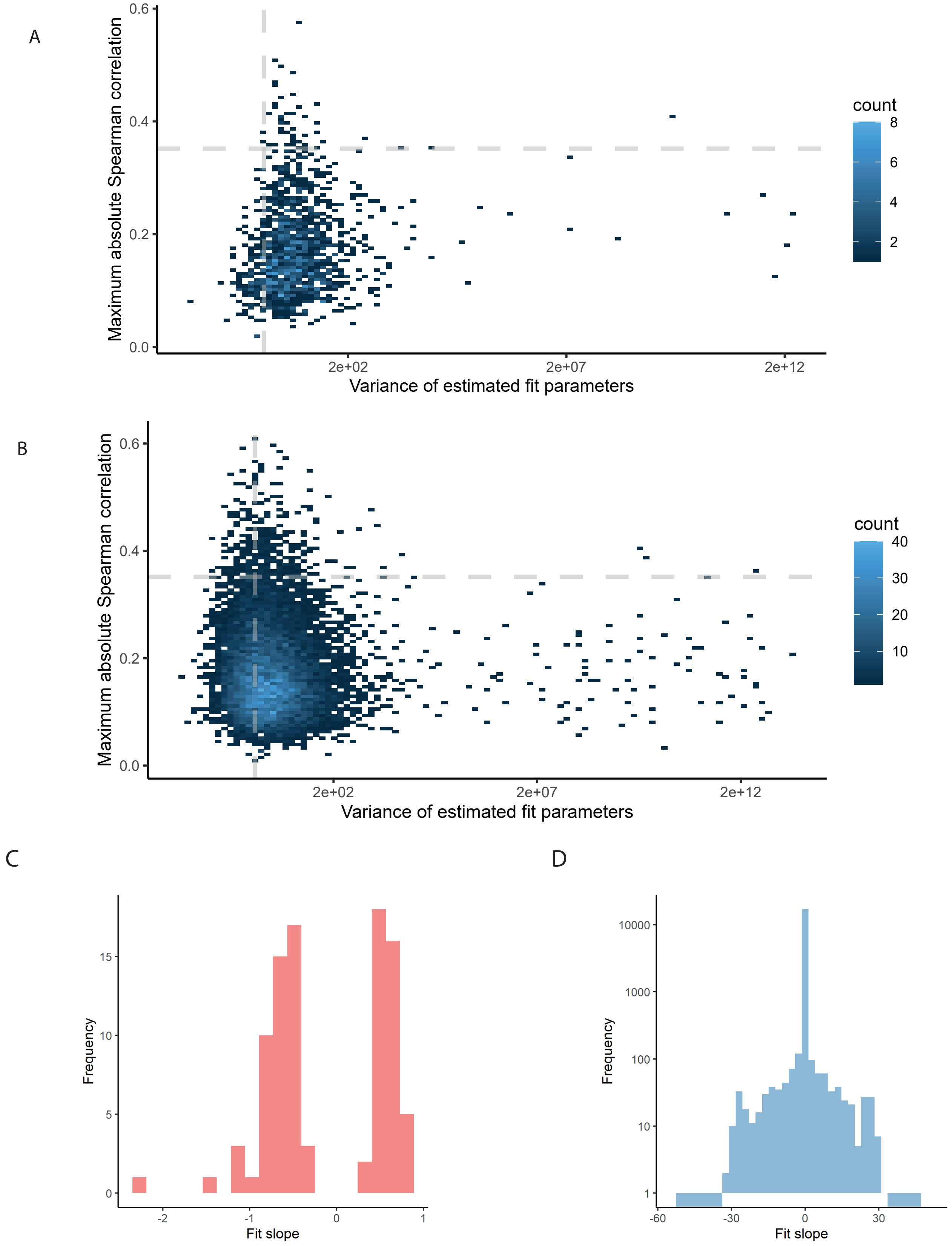

Appendix Figure S4: The distribution of coefficients resulting from the fitting of drug-gene similarity
a) The distribution of maximum absolute Spearman correlation coefficients and the variance of estimated fit parameters for the comparison of all drugs to STE2 is shown. The values found for the metabolome comparison of alpha factor to STE2 are shown with grey dashed lines, indicating that comparisons to the left of the dashed line have show a better fit to the logistic function than the alpha factor comparison, and comparisons above the horizontal line show a better correlation than the alpha factor comparison. b) The data represented in a is shown for all receptors. c) The distribution of maximum curve slopes is shown for logistic curve fitting of similarity between drug and overexpression metabolome profiles for drug-gene comparisons with fit qualities in the top 10% and maximum absolute correlation coefficients in the top 5%. d) The distribution of maximum curve slopes for logistic curve fitting of similarity between drug and overexpression metabolome profiles is shown for all comparisons.

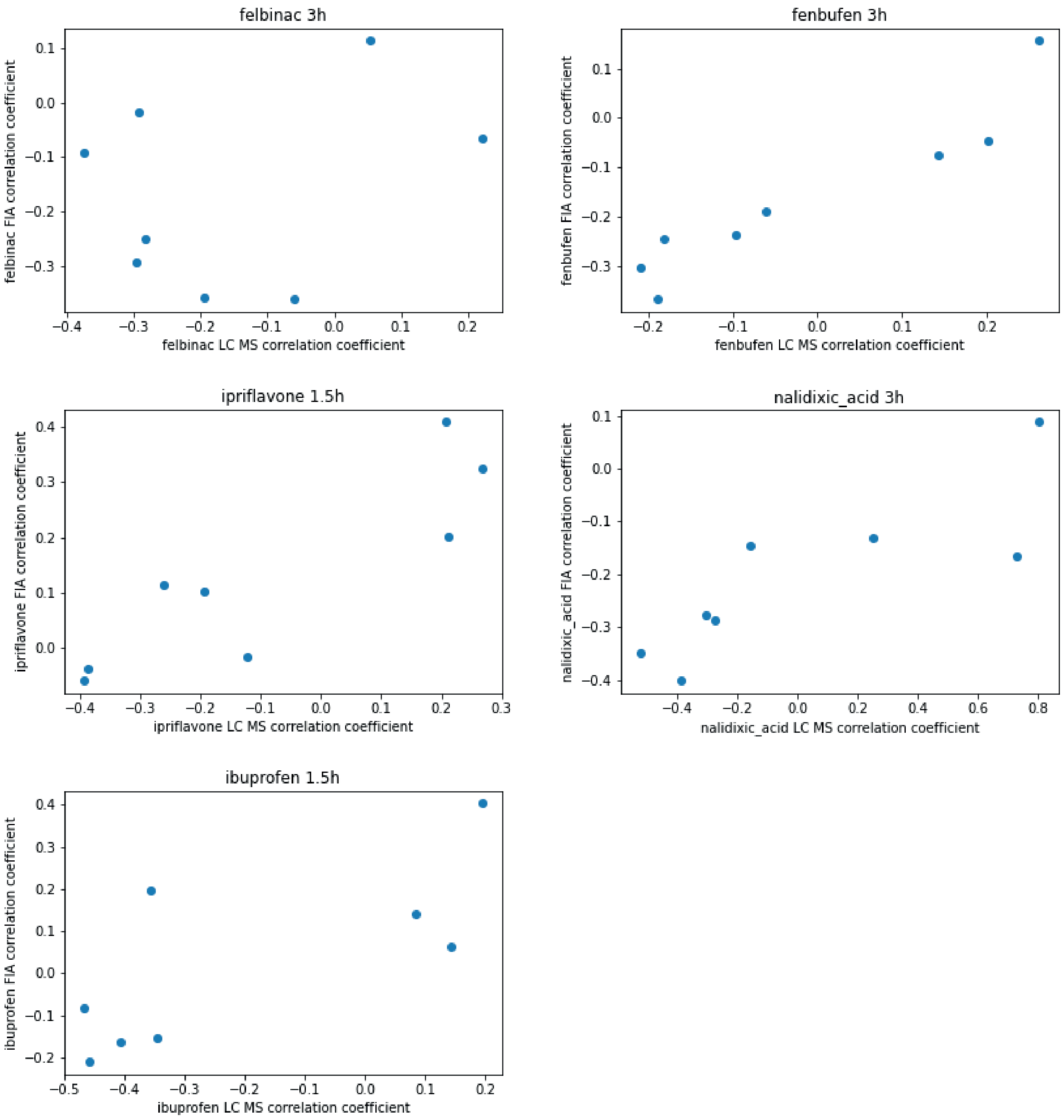

Appendix Figure S5: LC-MS analysis recaptures drug-gene relationships for working GPR1 antagonists
The Spearman correlation coefficients for the correlations between GPR1 overexpression conditions and the indicated drug treatments at the indicated time points as measured by flow-injection analysis are compared to the Pearson correlation coefficients for the same treatments as measured with liquid-chromatography mass spectrometry analysis.

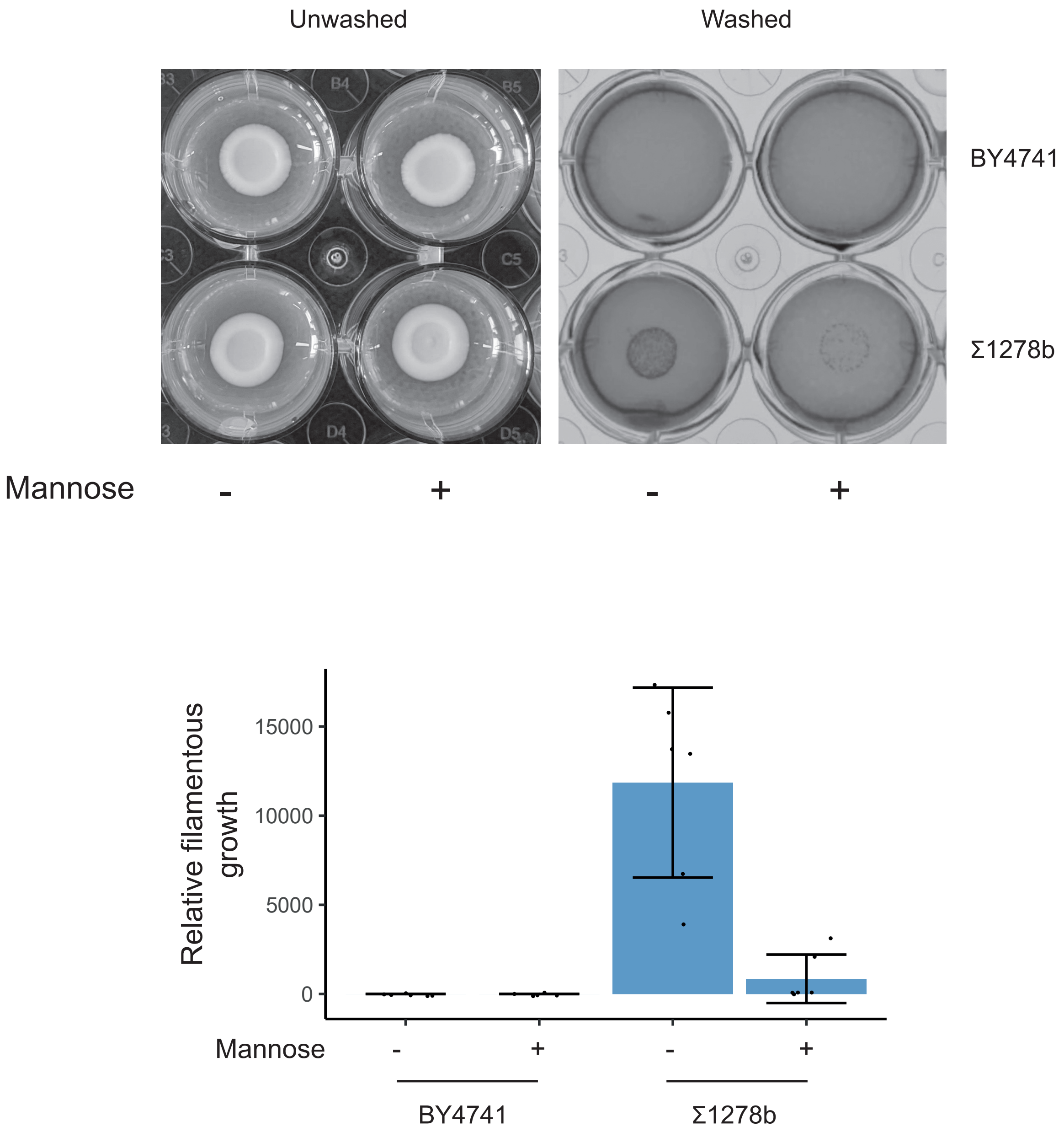

Appendix Figure S6: The effect of the known GPR1 antagonist mannose on filamentous growth
Representative images of BY4741 and Σ1278b yeast spotted onto media supplemented with either a water control or mannose (2% w/v). The colonies are shown both before and after washing in order to show filamentous growth. The relative quantified amount of filamentous growth for 6 technical replicates is shown with error bars representing standard deviation. Comparable results were obtained in three independent experiments.

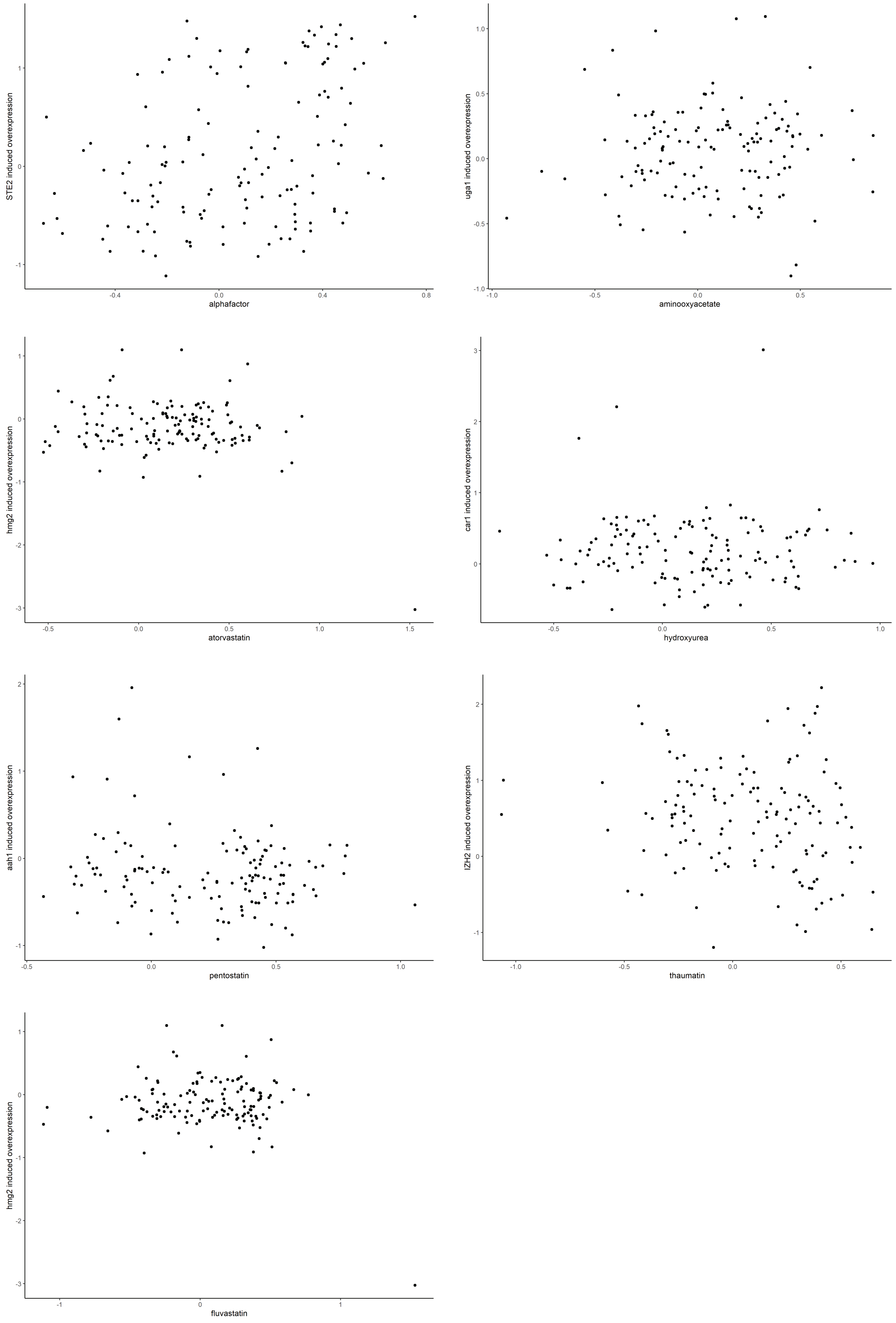

Appendix Figure S7: Bivariate scatterplots of drug-gene true positive pairs

The z-scores for true positive interactions as laid out in in Dataset EV 8 are represented where each point represents a metabolite.

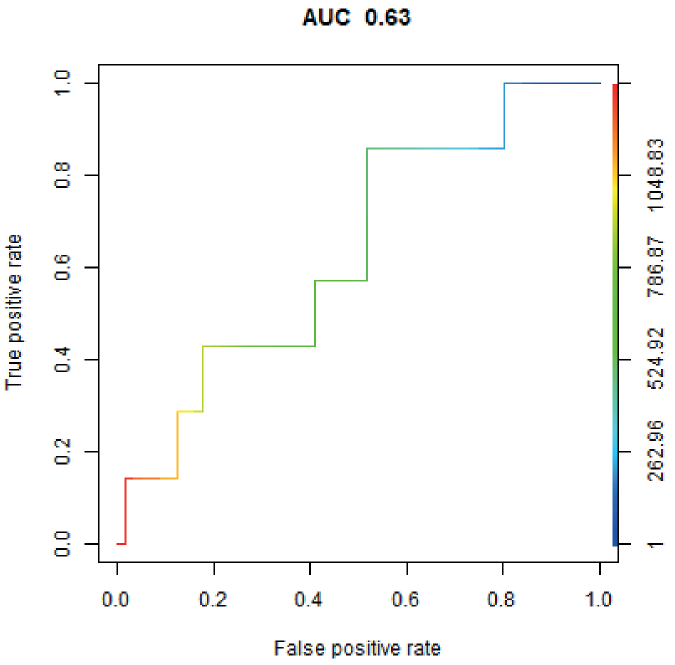

Appendix Figure S8: The recovery of true-positive interactions for uninduced YETI strains
A ROC curve representation of the recovery of true positives and false positives for the comparison of drug and mutant metabolome profiles by Pearson correlation. The area under the curve for the recover of the true-positives was 0.63.

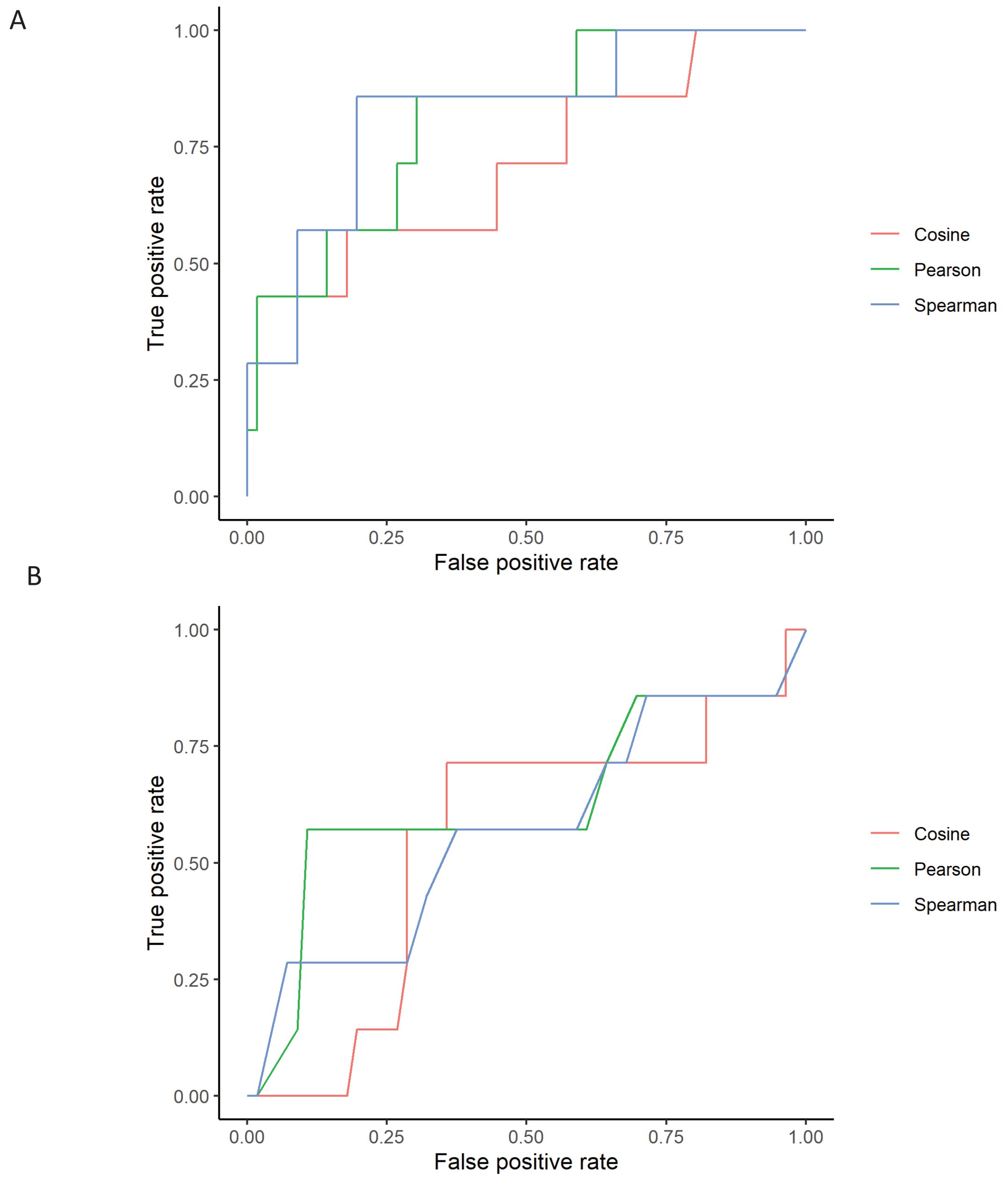

Appendix Figure S9: Recovery of true positives with altered ion inclusion criteria

a) ROC curves indicate the recovery of 7 true-positive drug-target interactions within the dataset compared to the number of false discoveries from the remaining 1280 drugs within the dataset. The recovery of expected interactions is shown using Spearman correlation, Pearon correlation, and cosine similarity. These analyses were performed for all shared ions annotated as a part of the KEGG library without restriction to only metabolites from the *S. cerevisiae* model. These similarity metrics allow for an area under the curve of 0.82, 0.81, and 0.71 for those similarity metrics respectively b) The same analysis as above is depicted, but for data where ions with a coefficient of variation of greater than 0.25 are discarded from the analysis. The areas under the curve for Spearman correlation, Pearson correlation, and cosine similarity were 0.57, 0.63, and 0.55 respectively.

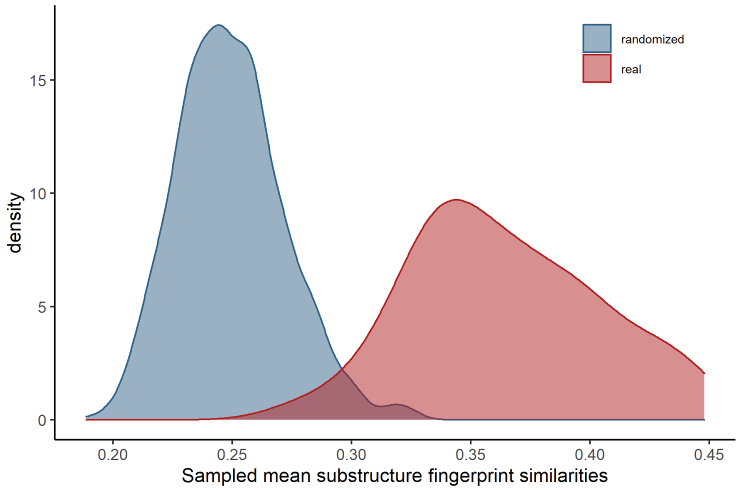

Appendix Figure S10: Drugs predicted to share same target share structural similarity
The mean structural similarity of the top 10 compounds for each target as represented in figure 4 is shown as a density plot, together with the same measure for 1000 scrambled iterations of the dataset.

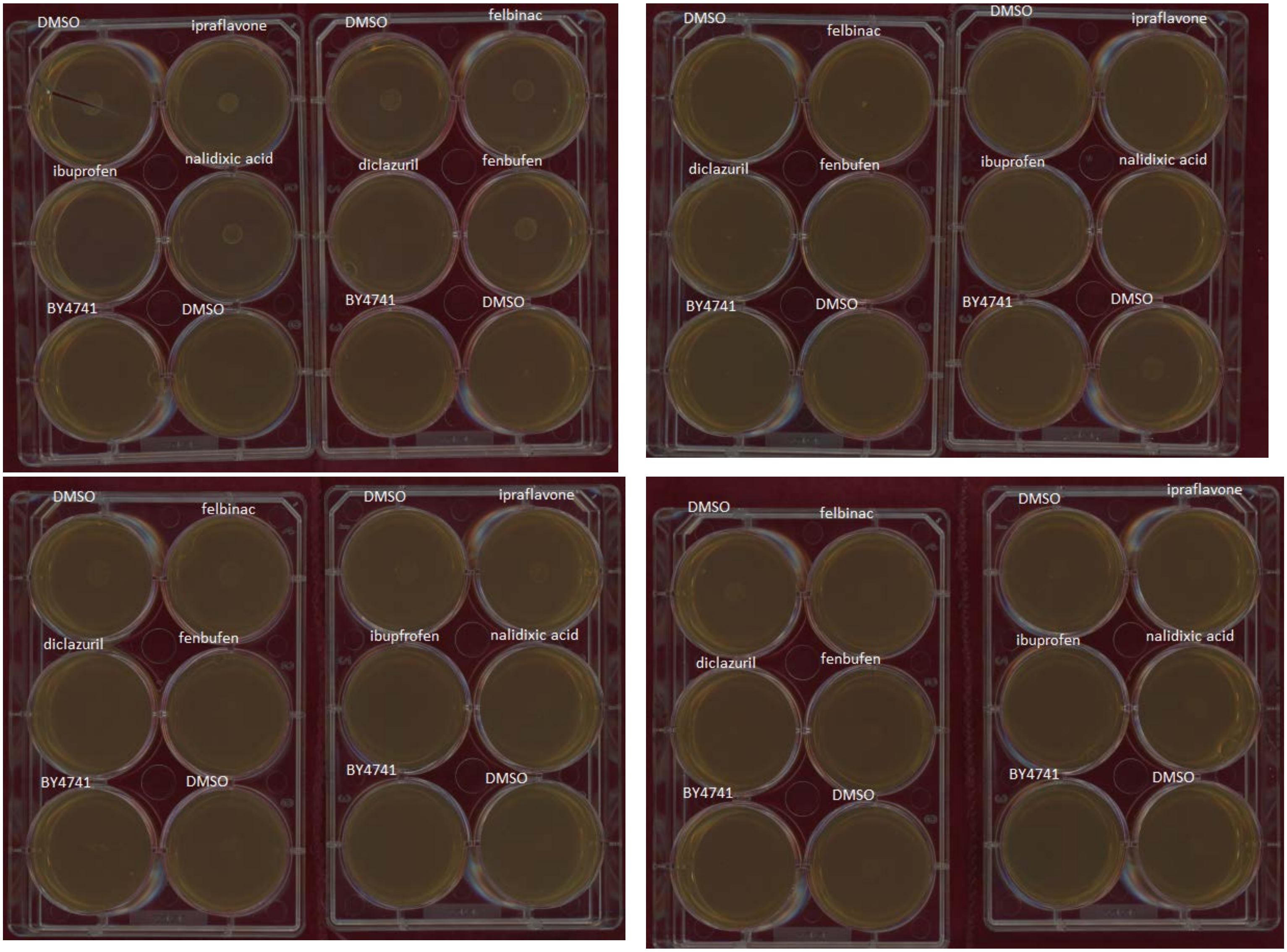

Apprendix Figure S11: Effect of predicted GPR1 antagonists on filamentous growth

Each image contains the results of filamentous growth assays with the indicated drug treatments performed on 1278b yeast except where specified otherwise

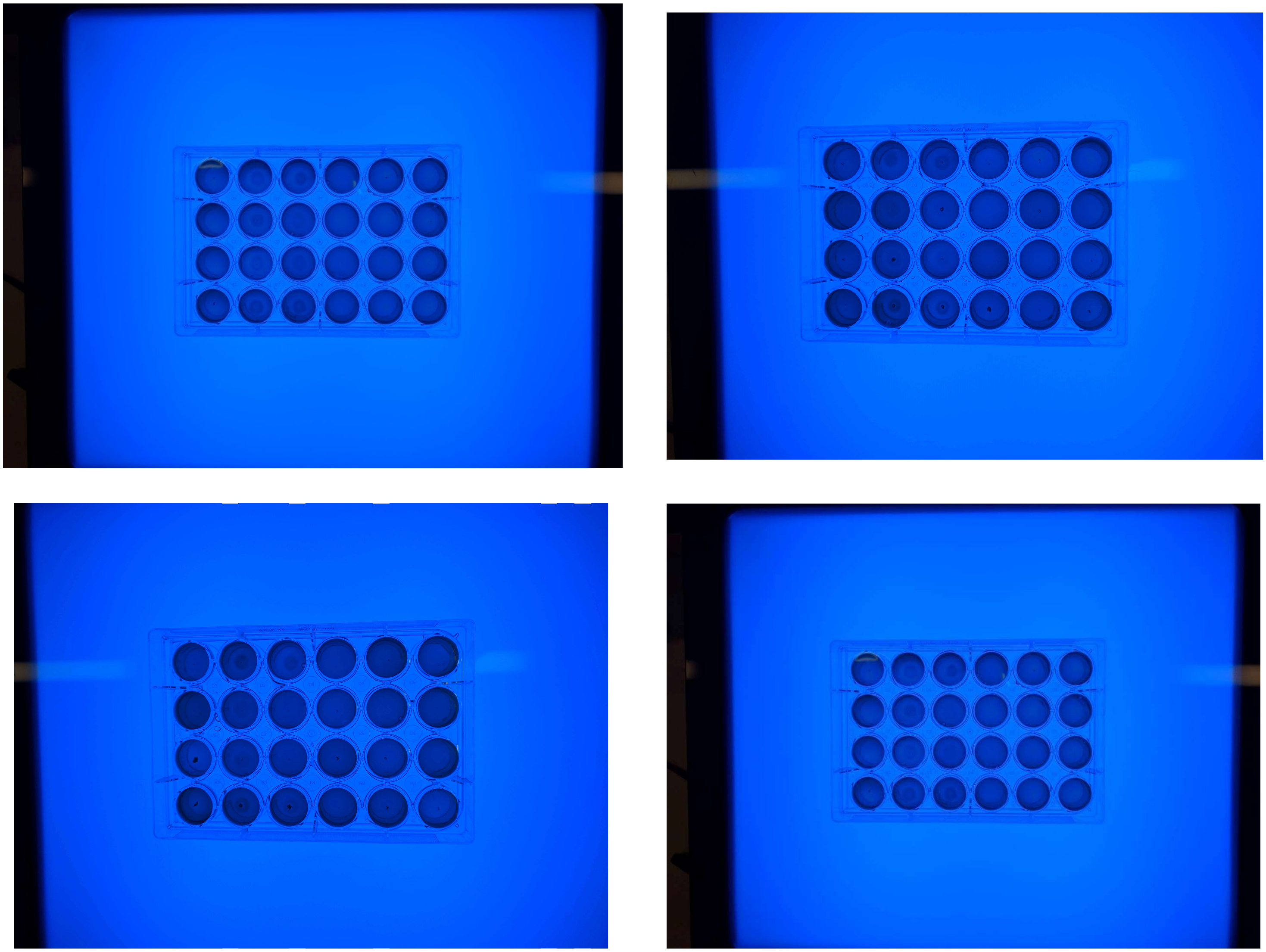

Appendix Figure S12: Dose-response of filamentous growth with ibuprofen treatment
Each column in the separate images contain 4 replicates of the same experiment. The column contents within each image are as follows: BY4741 yeast treated with DMSO, sigma1278b treated with DMSO; and sigma1278b treated with 1, 10, 20, and 50 micromolar ibuprofen.

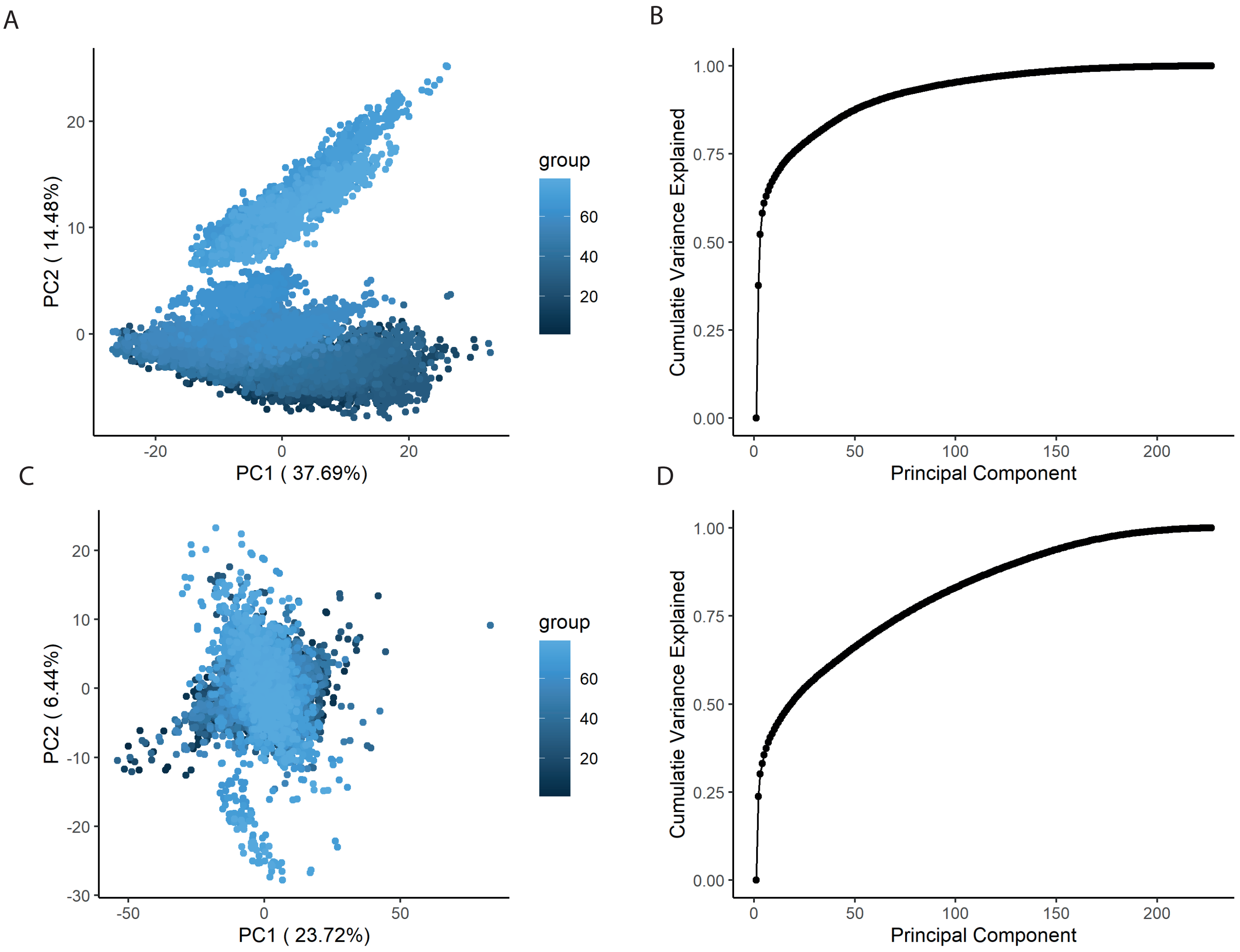

Appendix Figure S13: The effect of normalization of dataset variance
a) A principal component representation of the variance of the unnormalized mass spectrometry data for the main chemical screen and induced overexpression is represented. Colouring is based on the cultivation plate from which the samples were derived. b) The cumulative variance explained by the principal components is indicated for the unnormalized data represented in pannel a. c) A principal component representation of the variance of the normalized mass spectrometry data for the main chemical screen and induced overexpression is represented. Colouring is based on the cultivation plate from which the samples were derived. d) The cumulative variance explained by the principal components is indicated for the normalized data represented in pannel c.
