# Supplementary material for: High‐throughput metabolomics predicts drug–target relationships for eukaryotic proteins
Source: Mol Syst Biol. 2022 Feb 23;18(2):e10767. doi: 10.15252/msb.202110767 (PMC8864444; doi:10.15252/msb.202110767)
Supplement: Supplementary file 11 [file MSB-18-e10767-s007.pdf]

molecular systems biology

# High-throughput metabolomics predicts drug-target relationships for eukaryotic proteins

Duncan Holbrook-Smith, Stephan Durot, and Uwe Sauer
**DOI: 10.15252/msb.202110767**

*Corresponding author(s): Uwe Sauer (sauer@imsb.biol.ethz.ch)*



## Review Timeline:

| | |
|---|---|
| Submission Date: | 22nd Oct 21 |
| Editorial Decision: | 17th Nov 21 |
| Revision Received: | 6th Jan 22 |
| Editorial Decision: | 14th Jan 22 |
| Revision Received: | 18th Jan 22 |
| Accepted: | 21st Jan 22 |

*Editor: Maria Polychronidou*


## Transaction Report:

(Note: With the exception of the correction of typographical or spelling errors that could be a source of ambiguity, letters and reports are not edited. Depending on transfer agreements, referee reports obtained elsewhere may or may not be included in this compilation. Referee reports are anonymous unless the Referee chooses to sign their reports.)

Thank you again for submitting your work to Molecular Systems Biology. We have now heard back from the two reviewers who agreed to evaluate your study. As you will see below, the reviewers acknowledge that the study is a relevant contribution to the field. They raise however a series of concerns, which we would ask you to address in a revision.

I think that the reviewers' recommendations are clear and therefore there is no need to repeat the points listed below. All issues raised by the reviewers need to be satisfactorily addressed. Please contact me in case you would like to discuss in further detail any of the issues raised.

On a more editorial level, we would ask you to address the following points.

Reviewer #1:

This manuscript by Holbrook-Smith et al. seeks to identify drug-target interactions by identifying similarities in the metabolomic profiles of yeast treated with drugs and with inducible expression of drug targets. The article is a very nice proof of concept and will be of great interest to the drug development community. However, there are several issues which the authors should address to strengthen their manuscript:

1) The authors appear to focus on drugs that affect signaling which could fall short of the "promise of universal prediction of drug-target interactions" (page 3). Many drugs perturb metabolism (e.g., some of the classical chemotherapeutics like methotrexate). Can this method identify drugs that perturb metabolism?

2) On page 5, the authors use "eight different concentrations of the inducer... for a duration of 1.5 hours and 3 hours." Do the authors use data from both time points in subsequent analyses (e.g., Fig. 1)? It is unclear from the text whether both time points are used and/or necessary for drug-target prediction.

3) Page 5-6, the authors state that "disaccharides accumulated in GPR1 overexpression strains in conditions where less than 1 nM of inducer was added, and a decrease in disaccharide levels occurred in cases where more inducer was added (Fig S1). The accumulation of the disaccharide trehalose is a previously observed phenotype of GPR1 loss of function mutants32 that occurs due to its contribution to signaling through protein kinase A." My question is, why are disaccharides increased in the GPR1 strain treated with 0.1 nM inducer compared to untreated cells? Shouldn't the untreated cells act like a complete knockdown and therefore have greater disaccharide accumulation that 0.1 nM treated cells?

4) How reproducible are the metabolomics data? Have the authors run samples in biological replicates? If not, the authors should show reproducibility for a subset of the drugs treatments and/or yeast overexpression lines. Would filtering out metabolites that are not reproducibly measurable improve the drug-target predictions?

5) From the metabolomics data, the authors used 226 metabolites (selected from KEGG) out of 1,209 annotated. First, which of the 226 metabolites are most informative Are there metabolites which are poor information carriers and could potentially be removed from the analysis? Second, have the authors tried the drug-target prediction using the 1,209 annotated metabolites? Are there drug-target predictions which were missed by using only 226 KEGG metabolites?

6) Because the phenotypic readout is metabolism, I would expect that the media in which the yeast are grown will affect the prediction of drug-target relationships. Do the authors have any insight into how the media might affect the recovery of drug-target interactions, especially for inhibitors of metabolic enzymes (this is related to my comment above about metabolic inhibitors like methotrexate)?

Minor point: On page 7, the authors state "When the alpha factor peptide, an agonist for STE2, was included in these analyses it ranked in the top 1.6% of filtered comparisons for STE2 (Fig 2A)." It would be helpful if the authors could indicate which dot in Fig. 2A corresponds to alpha factor peptide.

Reviewer #2:

Summary

Holbrook-Smith and colleagues describe an approach for identifying novel drug-target interactions using metabolomics. They do this by generating metabolomic profiles following the addition of drugs, and the induction (or titration) of individual genes. They then compare drugs' and targets' metabolomic profiles to pair drugs with their ostensible target. They provide a detailed characterization of drugs targeting GPR1 through orthogonal screening and characterization of mechanism of action, and validate that their screening approach enriches known drug-target relationships.

General remarks

Understanding the on- and off-target effects of therapeutics is a perennial problem in drug and development. Several features of the presented work are particularly attractive for this problem including its high-throughput, compatibility with membrane-bound and soluble proteins, and high-dimensional read-out. Generating in vivo 'omic profiles following drug perturbations is an established strategy perhaps best exemplified by the Broad Institute's connectivity map effort: https://clue.io/cmap. The main conceptual advance of this work as compared to CMAP and other similar efforts is the integration of drug perturbation profiles with inducible genetic perturbations. Such comparisons provide a straight-forward path towards functional annotation if the target has a specific metabolomic fingerprint.

The general strategy appears sound but the manuscript is lacking crucial details that make it difficult to assess the overall merit and performance of the method. The functional characterization of GPR1 antagonists is clear, but I'm not convinced that any

other signals exist which would readily support functional characterization. A more thoughtful statistical treatment of the analyses would help along with a clearer dissection of specific (proximal to drug-protein interactions) versus non-specific (distal effects on GR, ESR, etc) effects.

Should the major concerns below be addressed, the manuscript would be of general interest for readers interested in chemical genomics and screening.

Major

1. The authors assert that "Although growth rate is known to exert a strong effect on the metabolome, after biomass normalization there was no appreciable increase in hit quality from growth rate normalization." I have a really hard time believing this unless the authors clearly demonstrate this point. Cycloheximide and likely many other drugs should perturb growth rate (GR) and/or the environmental stress response (ESR) resulting in metabolomic perturbations. The genetic perturbations themselves are known to induce a moderate ESR (32181581). Since these perturbations of global effects will be common across many drug perturbations they will tend to make perturbation profiles more similar and hence more difficult to readily pair for the purpose of functional annotation. One place to start here is looking at trehalose as a marker of the ESR and 1,3 BPG and orotate as a GR marker (19889834).

2. The authors' overall approach lacks a statistical foundation. This undermines some results but also detracts from the merits of this work as a scalable platform. First off, the author's assert that only 14% of drug perturbations result in more than a 2-fold change of metabolites. If most perturbations are just noise then they should not be compared to genetic perturbations. Generally, this will be innocent since comparisons to noise should not score highly, but it does limit interpretability of Figure 4C since each drug's most similar genes are summarized. To address this, the authors should carry out differential abundance testing of drug and genetic perturbations to remove inert perturbations.

3. The author's selection of hits is similarly non-statistical: "Comparisons with a covariance score indicating a fit in the top 5% of all comparisons were called as hits. Those hits were further filtered based on whether they had a maximum absolute spearman correlation coefficient in the top 5% of all of those measured across all comparisons." Please provide a more principled statistical solution for selecting hits.

4. "Raw ion intensities were normalized to counteract temporal drifts" please provide an appropriate citation or a more detailed treatment. The drug perturbation dataset itself is >1,000 samples. It would be nice to see the principal components plot (PC2 ~ PC1) and scree plot for the drug dataset to better understand how much variation is due to specific drug-protein interactions, downstream impacts on global effects, or lingering batch effects.

Minor

1. Figure 1B - What accounts for the pronounced structure in this matrix?

2. Figure 2B - Please show two bars rather than a difference. The difference could be very different things based on the number of metabolites changing in each mutant strain.

3. The authors indicate that ZEV strains are loss-of-function in the absence of estradiol. This is no longer thought to be true (34096681). In fact, many essential genes grow fine in the absence of estradiol if they are low copy.

4. Figure S1 - Why are only 8 metabolites shown for each dose-response; how were they selected?

5. Figure S4 - It looks like many fits are pathological (extreme residual variance); please elaborate.

6. For Figure 4B, please provide bivariate scatterplots of drug-gene true positive pairs. It would be nice to know whether the large discrepancies in AUC between Pearson, Spearman and cosine-similarity are due to the small number of TPs or whether some distance measures are more appropriate than others (for example, high Pearson correlation driven by single metabolite should favor the other distance measures).

7. The description of z-scores is confusing. It would be best to just indicate how x, mu, and sigma are estimated.

8. "overexpression and time point dose response, the data was scaled between 0 and 1." How is this done? The normal cdf?

9. Figure 1D - "scaled correlation" does not seem like appropriate terminology

10. Figure 1E - I don't think this adds anything

Firstly, we would like to express our sincere appreciation of the comments from our reviewers. Below you will find their comments paired with our responses to them. We have also indicated where within the revised manuscript we have made changes to address those the matters raise within those comments.

*Reviewer #1:*

*This manuscript by Holbrook-Smith et al. seeks to identify drug-target interactions by identifying similarities in the metabolomic profiles of yeast treated with drugs and with inducible expression of drug targets. The article is a very nice proof of concept and will be of great interest to the drug development community. However, there are several issues which the authors should address to strengthen their manuscript:*

*1) The authors appear to focus on drugs that affect signaling which could fall short of the "promise of universal prediction of drug-target interactions" (page 3). Many drugs perturb metabolism (e.g., some of the classical chemotherapeutics like methotrexate). Can this method identify drugs that perturb metabolism?*

Given that our readout are metabolite levels, the method is by definition expected to perform even better on metabolic drugs. We included the metabolic drugs atorvastatin, hydroxyurea, aminooxyacetate, pentostatin, and fluvastatin within our set of positive controls described in figure 4b. These drugs were recovered with similar efficacy as the signaling drugs within the set of controls. The text has been amended on page 4 to make this clearer.

*2) On page 5, the authors use "eight different concentrations of the inducer... for a duration of 1.5 hours and 3 hours." Do the authors use data from both time points in subsequent analyses (e.g., Fig. 1)? It is unclear from the text whether both time points are used and/or necessary for drug-target prediction.*

The data for both time points are indeed used for subsequent analysis. Both time points are included in order to allow for broader recovery of patterns of metabolome similarities from drugs which may mimic the effect of rather shorter or longer term overexpression. The text on page 7 has been amended to clarify that the data from both time points are used.

*3) Page 5-6, the authors state that "disaccharides accumulated in GPR1 overexpression strains in conditions where less than 1 nM of inducer was added, and a decrease in disaccharide levels occurred in cases where more inducer was added (Fig S1). The accumulation of the disaccharide trehalose is a previously observed phenotype of GPR1 loss of function mutants32 that occurs due to its contribution to signaling through protein kinase A." My question is, why are disaccharides increased in the GPR1 strain treated with 0.1 nM inducer compared to untreated cells? Shouldn't the untreated cells act like a complete knockdown and therefore have greater disaccharide accumulation that 0.1 nM treated cells?*

We would expect both untreated and very low concentration treatments to have an accumulation of disaccharide. The low concentration treatments with estradiol (less than 10 nM) were shown in other cases to be insufficient to induce overexpression (PMID 23275543). For this reason, we would expect those low concentrations of estradiol to be equivalent to cause an increase in disaccharide levels. However, the relative increase in disaccharide in the 0.1 nM treatment compared to the 0 nM treatment is due to experimental noise in one of the 0 nM estradiol concentration data points. Despite this experimental noise, the approach was able to recover small molecules that perturb disaccharide levels in a GPR1 dependent manner,

as is highlighted in Figure 2C.

*4) How reproducible are the metabolomics data? Have the authors run samples in biological replicates? If not, the authors should show reproducibility for a subset of the drugs treatments and/or yeast overexpression lines. Would filtering out metabolites that are not reproducibly measurable improve the drug-target predictions?*

As stated in the "Metabolite extraction" segment of the Materials and Methods section, both the Prestwick and the YETI library screens were done in biological quadruplicates in separate microtiter plates, each of which was then analyzed as technical duplicates. For the dose-response overexpression experiments, biological replicates were cultivated in separate wells of the same plate, but were inoculated from a precultures that were also cultivated in separate wells allowing for many generations separation between the replicates. In addition, the results obtained from the comparison of drug to dose-response overexpression were confirmed in separate experiments using a separate methodology and very similar results were obtained for 4 out of the 5 drugs that were tested (Appendix Figure S5). This demonstrates reproducibility both in the experimental collection of samples, as well as the analytical treatment of those samples.

We did not filter ions on the basis of whether they showed higher or lower coefficients of variation between replicates. Although a particular ion may not appear to be measured with consistent levels across replicates, a particular treatment can cause large robust changes that can have explanatory power due to that change being very characteristic of a particular drug or overexpression treatment. Missing those few but large changes was in general not a trade-off we wished to pursue.

*5) From the metabolomics data, the authors used 226 metabolites (selected from KEGG) out of 1,209 annotated. First, which of the 226 metabolites are most informative Are there metabolites which are poor information carriers and could potentially be removed from the analysis? Second, have the authors tried the drug-target prediction using the 1,209 annotated metabolites? Are there drug-target predictions which were missed by using only 226 KEGG metabolites?*

Fundamentally we decided to restrict the considered metabolites to the 226 KEGG metabolites in order to reduce the potential for the misannotation of drugs from the Prestwick chemical library as endogenous metabolites that change as a result of drug treatments. Although steps were taken to limit this possibility, such as filtering out apparent drug derived ions based on comparison to measurement of the drugs themselves, we opted to be conservative and restrict ourselves to metabolites we would expect to observe in *S. cerevisiae.*

However, with the comparisons that were made in Figure 4, we can assess more systematically how using a larger set of ions (the whole KEGG collections) affect the recovery of true positives. Our analyses represented in appendix figure S9 showed that the inclusion of the larger set of ions did not increase the maximum recovery rate of the positive control interactions compared to the more restrictive approach, although the performance of Spearman correlation is improved when more metabolites are included.

In order to test whether restricting the analysis to the ions which are poor carriers of information, we excluded ions with median coefficients of variation for their ion intensity of greater than 0.25. With this restriction, the recovery of positive controls was significantly

reduced as is shown in appendix figure S9. This is likely due to some ions that are very informative for specific targets being measured somewhat noisily.

*6) Because the phenotypic readout is metabolism, I would expect that the media in which the yeast are grown will affect the prediction of drug-target relationships. Do the authors have any insight into how the media might affect the recovery of drug-target interactions, especially for inhibitors of metabolic enzymes (this is related to my comment above about metabolic inhibitors like methotrexate)?*

This is an excellent point, and was an important consideration when the chemical screen was devised. Especially for drug targets related to metabolism, there are clear trade-offs between different media conditions in terms of the successful interrogation of specific targets. For example, in this study the amino acid sensor GAP1 was used as a target. It seems reasonable that if we hope to find inhibitors for GAP1 that our chances will be higher if we screen for metabolome effects in the presence of exogenous amino acids (as was done in this study). Conversely, it could be harder to identify antagonists for enzymes whose activities are inhibited in the presence of exogenous amino acids since the difference between the inhibition of the enzyme by regulatory or pharmacological means will be less clear. For this reason, not every target will be ideally recovered by every screening condition. This is, however, a common limitation of functional genomics, chemical genomics, and conventional chemical screens. In all cases, experimental conditions must be chosen that are the same across all genes/drugs. These choices increase the recovery of information for some targets at the cost of others, but still allow for broad surveys of general utility. Our choice of condition was made based on the general targets we were considering in order to increase our chances of success. A discussion of this topic has been added to page 17.

*Minor point: On page 7, the authors state "When the alpha factor peptide, an agonist for STE2, was included in these analyses it ranked in the top 1.6% of filtered comparisons for STE2 (Fig 2A)." It would be helpful if the authors could indicate which dot in Fig. 2A corresponds to alpha factor peptide.*

A yellow star has been added to indicate the position of the absolute maximum correlation between alpha factor and STE2 in order to clarify the figure. The original figure is based on only compounds that exist within the Prestwick library, so this point was not originally depicted within the figure.

*Reviewer #2:*

*Summary*

*Holbrook-Smith and colleagues describe an approach for identifying novel drug-target interactions using metabolomics. They do this by generating metabolomic profiles following the addition of drugs, and the induction (or titration) of individual genes. They then compare drugs' and targets' metabolomic profiles to pair drugs with their ostensible target. They provide a detailed characterization of drugs targeting GPR1 through orthogonal screening and characterization of mechanism of action, and validate that their screening approach enriches known drug-target relationships.*

*General remarks*

*Understanding the on- and off-target effects of therapeutics is a perennial problem in drug and development. Several features of the presented work are particularly attractive for this problem including its high-throughput, compatibility with membrane-bound and soluble proteins, and high-dimensional read-out. Generating in vivo 'omic profiles following drug perturbations is an established strategy perhaps best exemplified by the Broad Institute's connectivity map effort: [https://clue.io/cmap](https://clue.io/cmap). The main conceptual advance of this work as compared to CMAP and other similar efforts is the integration of drug perturbation profiles with inducible genetic perturbations. Such comparisons provide a straight-forward path towards functional annotation if the target has a specific metabolomic fingerprint.*

*The general strategy appears sound but the manuscript is lacking crucial details that make it difficult to assess the overall merit and performance of the method. The functional characterization of GPR1 antagonists is clear, but I'm not convinced that any other signals exist which would readily support functional characterization. A more thoughtful statistical treatment of the analyses would help along with a clearer dissection of specific (proximal to drug-protein interactions) versus non-specific (distal effects on GR, ESR, etc) effects.*

*Should the major concerns below be addressed, the manuscript would be of general interest for readers interested in chemical genomics and screening.*

*Major*

*1. The authors assert that "Although growth rate is known to exert a strong effect on the metabolome, after biomass normalization there was no appreciable increase in hit quality from growth rate normalization." I have a really hard time believing this unless the authors clearly demonstrate this point. Cycloheximide and likely many other drugs should perturb growth rate (GR) and/or the environmental stress response (ESR) resulting in metabolomic perturbations. The genetic perturbations themselves are known to induce a moderate ESR (32181581). Since these perturbations of global effects will be common across many drug perturbations they will tend to make perturbation profiles more similar and hence more difficult to readily pair for the purpose of functional annotation. One place to start here is looking at trehalose as a marker of the ESR and 1,3 BPG and orotate as a GR marker (19889834).*

We believe the normalization we performed was sufficient to control for the effect of growth within the data. Within the normalized data, orotate and trehalose (annotated as disaccharide) showed either no correlation or a weak negative correlation to the relative growth rates (-0.01

and -0.12 respectively). These low residual relationships between those ion intensities and growth rate in the normalized data demonstrates that added normalization of that basis would add little in terms of removing unwanted variation. We did not annotate 1,3 BPG, so we cannot comment on it specifically.

*2. The authors' overall approach lacks a statistical foundation. This undermines some results but also detracts from the merits of this work as a scalable platform. First off, the author's assert that only 14% of drug perturbations result in more than a 2-fold change of metabolites. If most perturbations are just noise then they should not be compared to genetic perturbations. Generally, this will be innocent since comparisons to noise should not score highly, but it does limit interpretability of Figure 4C since each drug's most similar genes are summarized. To address this, the authors should carry out differential abundance testing of drug and genetic perturbations to remove inert perturbations.*

We respectfully disagree with the assessment that our approach lacks statistic foundation. Through the cultivation of drug-treated cells in biological quadruplicate in separate plates, we were able to compare relative metabolite levels for each drug treatment in comparison to the rest of those within that batch. This allowed for the z-scoring and t-test based p-value determination for each drug and metabolite while limiting the impacts of batch effects giving a clear statistical basis for which metabolites change and which do not for each treatment. Although a relatively small proportion of drug treatments cause 2 fold or greater changes in metabolome profiles, this does not imply by any means that those conditions do not contain meaningful information. In fact, only 25 drug treatments out of 1280 have one or fewer metabolites that show relative ion intensities that are different from the rest of the plate with a p-value of less than 0.05 suggesting that almost all of the drug treatments cause some significant change in metabolite levels.

To illustrate this point and address our reviewers comment with respect to figure 4C, we have repeated the analysis with drugs and genetic perturbations limited to ones where at least five metabolites change a p-value of less than 0.05. The new version of Figure 4C contains this representation. The removal of these drugs did not cause a large change in the distribution of hits across the genes in the study. This demonstrates that the broad distribution of drug-target predictions is not a result of the inclusion of drugs that cause few or no significant changes in the metabolome.

*3. The author's selection of hits is similarly non-statistical: "Comparisons with a covariance score indicating a fit in the top 5% of all comparisons were called as hits. Those hits were further filtered based on whether they had a maximum absolute spearman correlation coefficient in the top 5% of all of those measured across all comparisons." Please provide a more principled statistical solution for selecting hits.*

We respectfully disagree that our method for hit selection is non-statistical. Both the maximum similarity and fit quality are metrics that we expect to be relevant for identifying relationships between drugs and genes. For any screening approach, the discrimination between hits and misses relies on some threshold value. The values we originally applied reflected fit qualities and similarity scores were reasonable based on the alpha-factor to STE2 comparison and reflect a common cutoff applied in biological sciences. However, we decided to alter our hit calling approach in order to explicitly leverage the alpha factor to STE2 comparison. In our revision we have opted to require hits to show a maximum absolute correlation that is at least as large as that observed for the comparison of the positive control alpha factor to STE2 comparison. With respect to the logistic fit quality, we now have two

criteria. Firstly, we now require that the inflection point of the logistic fit falls within the range of concentrations of inducer that were added so as to filter out pathological fits that do not capture the expected dose-response behaviour. Secondly, comparisons that pass that criteria, hits must possess a fit covariance in the top 10% in terms of fit quality**.** This relative cutoff for fit quality allows for the most promising hits across the different targets to be examined, while the absolute cutoff requires that the relationship contain a correlation that is as strong or stronger than a known interaction. While the 10% cutoff is somewhat arbitrary, the function of that parameter is to ensure that the recovered hits show some broad dose-dependence, but the eventual quality of a lead is not tremendously sensitive to whether the fit is perfect or not.

*4. "Raw ion intensities were normalized to counteract temporal drifts" please provide an appropriate citation or a more detailed treatment. The drug perturbation dataset itself is >1,000 samples. It would be nice to see the principal components plot (PC2 ~ PC1) and scree plot for the drug dataset to better understand how much variation is due to specific drug-protein interactions, downstream impacts on global effects, or lingering batch effects.*

A more detailed description of temporal drift normalization has been included in the methods section. The requested principal component and scree plots depicting the variation in the data before and after normalization have been included in Appendix Figure S13. They show that within the unnormalized data there was indeed separation of batches, but that after normalization that separation is dramatically reduced. In addition, the normalized data requires a larger number of components in order to describe the variance in the data as is shown in the cumulative variance plot.

*Minor*

*1. Figure 1B - What accounts for the pronounced structure in this matrix?*

Along the y axis the various mutants with different concentrations and treatment durations of estradiol are presented in series, and without clustering. Conversely, the drugs along the x axis were clustered. This together with systematic trends in correlation results in the structure that is apparent within the data.

*2. Figure 2B - Please show two bars rather than a difference. The difference could be very different things based on the number of metabolites changing in each mutant strain.*

Figure 2B has been altered to conform with the reviewer request.

*3. The authors indicate that ZEV strains are loss-of-function in the absence of estradiol. This is no longer thought to be true (34096681). In fact, many essential genes grow fine in the absence of estradiol if they are low copy.*

The text has been updated to reflect that although the expression system is relatively tight, that for low copy genes the strains may not fully resemble a loss-of-function.

*4. Figure S1 - Why are only 8 metabolites shown for each dose-response; how were they selected?*

The metabolites selected in these plots are chosen based on two criteria. Firstly, they were selected based on whether they showed significant differences between the control and overexpression strains. Secondly, they were chosen to provide some concrete examples of which sorts of metabolites change in abundance with respect to the strength of induction as well as the scale and nature of that dose-dependence.

*5. Figure S4 - It looks like many fits are pathological (extreme residual variance); please elaborate.*
As described above in the answer to major point 3, fits with an inflection point outside of the range of concentrations were originally not discarded. These fits generally lead to a very poor description of the data, resulting in the extreme residual variances that are observed. These fits have now been filtered out of consideration, and the picture is now much cleaner as can be seen in the revised supplemental figure 4. Whereas previously a significant number of fits with a variance of 2E12 or greater were seen, now for a representation that includes all 6 genes in the dataset the maximum variances are less that 2E12 and fewer in number. For remaining high variance fits, the vast majority of them are likely due to their showing maximum slopes that are an order of magnitude greater than what is observed within other fits in the dataset.

*6. For Figure 4B, please provide bivariate scatterplots of drug-gene true positive pairs. It would be nice to know whether the large discrepancies in AUC between Pearson, Spearman and cosine-similarity are due to the small number of TPs or whether some distance measures are more appropriate than others (for example, high Pearson correlation driven by single metabolite should favor the other distance measures).*

These representations are included in appendix figure S7. As the reviewer predicted, the strength of Pearson correlation which allows it to perform better than Spearman or cosine similarity is largely due to relatively large changes in few metabolites specifically associated with the drug's mode of action driving the relationship in some cases.

*7. The description of z-scores is confusing. It would be best to just indicate how x, mu, and sigma are estimated.*

A thorough description has been included within the relevant methods section.

*8. "overexpression and time point dose response, the data was scaled between 0 and 1." How is this done? The normal cdf?*

This was performed using the MinMaxScaler function from scikkit-learn. This information has been included in the relevant methods section.

*9. Figure 1D - "scaled correlation" does not seem like appropriate terminology*

The language in the plot has been changed to "Normalized correlation" with an expanded description within the figure caption, and the methods section to make it clear that this refers to min-max scaled correlations.

*10. Figure 1E - I don't think this adds anything*

This figure has been moved to the appendix in order to streamline the paper.

## 1st Revision - Editorial Decision 14th Jan 2022

Thank you for sending us your revised manuscript. We have now evaluated your revised study and we think that the performed revisions have satisfactorily addressed the issues raised by the reviewers. As such I am glad to inform your that we can soon proceed with accepting the study for publication. Before we proceed with the formal acceptance, we would ask you to address some minor editorial issues listed below.

## 2nd Authors' Response to Reviewers 18th Jan 2022

The authors have made all requested editorial changes.

## Accepted 21st Jan 2022

Thank you again for sending us your revised manuscript. We are now satisfied with the modifications made and I am pleased to inform you that your paper has been accepted for publication.

EMBO PRESS

YOU MUST COMPLETE ALL CELLS WITH A PINK BACKGROUND ↓

PLEASE NOTE THAT THIS CHECKLIST WILL BE PUBLISHED ALONGSIDE YOUR PAPER


Corresponding Author Name: Uwe Sauer



Journal Submitted to: Molecular Systems Biology

Manuscript Number: MSB-2021-10767


USEFUL LINKS FOR COMPLETING THIS FORM

http://www.antibodypedia.com
http://1degreebio.org
http://www.equator-network.org/reporting-guidelines/improving-bioscience-research-repor
http://grants.nih.gov/grants/olaw/olaw.htm
http://www.mrc.ac.uk/Ourresearch/Ethicsresearchguidance/Useofanimals/index.htm
http://ClinicalTrials.gov
http://www.consort-statement.org
http://www.consort-statement.org/checklists/view/32-consort/66-title
http://www.equator-network.org/reporting-guidelines/reporting-recommendations-for-tumo
http://datadryad.org
http://figshare.com
http://www.ncbi.nlm.nih.gov/gap
http://www.ebi.ac.uk/ega
http://biomodels.net/
http://biomodels.net/miriam/
http://jjj.biochem.sun.ac.za
https://osp.od.nih.gov/biosafety-biosecurity-and-emerging-biotechnology/
http://www.selectagents.gov/

## Reporting Checklist For Life Sciences Articles (Rev. June 2017)

This checklist is used to ensure good reporting standards and to improve the reproducibility of published results. These guidelines are consistent with the Principles and Guidelines for Reporting Preclinical Research issued by the NIH in 2014. Please follow the journal's authorship guidelines in preparing your manuscript.

### A- Figures

### 1. Data

### The data shown in figures should satisfy the following conditions:

- the data were obtained and processed according to the field's best practice and are presented to reflect the results of the experiments in an accurate and unbiased manner.
- figure panels include only data points, measurements or observations that can be compared to each other in a scientifically meaningful way.
- graphs include clearly labeled error bars for independent experiments and sample sizes. Unless justified, error bars should not be shown for technical replicates.
- if n< 5, the individual data points from each experiment should be plotted and any statistical test employed should be justified
- Source Data should be included to report the data underlying graphs. Please follow the guidelines set out in the author ship guidelines on Data Presentation.

### 2. Captions

### Each figure caption should contain the following information, for each panel where they are relevant:

- a specification of the experimental system investigated (eg cell line, species name).
- the assay(s) and method(s) used to carry out the reported observations and measurements
- an explicit mention of the biological and chemical entity(ies) that are being measured.
- an explicit mention of the biological and chemical entity(ies) that are altered/varied/perturbed in a controlled manner.

- the exact sample size (n) for each experimental group/condition, given as a number, not a range;
- a description of the sample collection allowing the reader to understand whether the samples represent technical or biological replicates (including how many animals, litters, cultures, etc.).
- a statement of how many times the experiment shown was independently replicated in the laboratory.
- definitions of statistical methods and measures:
  - common tests, such as t-test (please specify whether paired vs. unpaired), simple $\chi^2$ tests, Wilcoxon and Mann-Whitney tests, can be unambiguously identified by name only, but more complex techniques should be described in the methods section;
  - are tests one-sided or two-sided?
  - are there adjustments for multiple comparisons?
  - exact statistical test results, e.g., P values = x but not P values < x;
  - definition of 'center values' as median or average;
  - definition of error bars as s.d. or s.e.m.

Any descriptions too long for the figure legend should be included in the methods section and/or with the source data.

**In the pink boxes below, please ensure that the answers to the following questions are reported in the manuscript itself. Every question should be answered. If the question is not relevant to your research, please write NA (non applicable). We encourage you to include a specific subsection in the methods section for statistics, reagents, animal models and human subjects.**

### B- Statistics and general methods

Please fill out these boxes ↓ (Do not worry if you cannot see all your text once you press return)

| | |
|---|---|
| 1.a. How was the sample size chosen to ensure adequate power to detect a pre-specified effect size? | Effect size was not specified prior to the analysis. However, at least three biological replicates were completed for each data point. |
| 1.b. For animal studies, include a statement about sample size estimate even if no statistical methods were used. | NA |
| 2. Describe inclusion/exclusion criteria if samples or animals were excluded from the analysis. Were the criteria pre-established? | NA |
| 3. Were any steps taken to minimize the effects of subjective bias when allocating animals/samples to treatment (e.g. randomization procedure)? If yes, please describe. | NA |
| For animal studies, include a statement about randomization even if no randomization was used. | NA |
| 4.a. Were any steps taken to minimize the effects of subjective bias during group allocation or/and when assessing results (e.g. blinding of the investigator)? If yes please describe. | NA |
| 4.b. For animal studies, include a statement about blinding even if no blinding was done | NA |
| 5. For every figure, are statistical tests justified as appropriate? | Yes. |
| Do the data meet the assumptions of the tests (e.g., normal distribution)? Describe any methods used to assess it. | Yes. Normality was asses through Shapiro-Wilk normality tests where appropriate. |
| Is there an estimate of variation within each group of data? | Estimates of variation are described within the relevant figure captions. |

| | |
|---|---|
| Is the variance similar between the groups that are being statistically compared? | Heteroscedastic approaches were used for p-value determination to remove any assumption of equal variance between groups. |

## C- Reagents

| | |
|---|---|
| 6. To show that antibodies were profiled for use in the system under study (assay and species), provide a citation, catalog number and/or clone number, supplementary information or reference to an antibody validation profile. e.g., Antibodypedia (see link list at top right), 1DegreeBio (see link list at top right). | NA |
| 7. Identify the source of cell lines and report if they were recently authenticated (e.g., by STR profiling) and tested for mycoplasma contamination. | NA |

* for all hyperlinks, please see the table at the top right of the document

## D- Animal Models

| | |
|---|---|
| 8. Report species, strain, gender, age of animals and genetic modification status where applicable. Please detail housing and husbandry conditions and the source of animals. | NA |
| 9. For experiments involving live vertebrates, include a statement of compliance with ethical regulations and identify the committee(s) approving the experiments. | NA |
| 10. We recommend consulting the ARRIVE guidelines (see link list at top right) (PLoS Biol. 8(6), e1000412, 2010) to ensure that other relevant aspects of animal studies are adequately reported. See author guidelines, under 'Reporting Guidelines'. See also: NIH (see link list at top right) and MRC (see link list at top right) recommendations. Please confirm compliance. | NA |

## E- Human Subjects

| | |
|---|---|
| 11. Identify the committee(s) approving the study protocol. | NA |
| 12. Include a statement confirming that informed consent was obtained from all subjects and that the experiments conformed to the principles set out in the WMA Declaration of Helsinki and the Department of Health and Human Services Belmont Report. | NA |
| 13. For publication of patient photos, include a statement confirming that consent to publish was obtained. | NA |
| 14. Report any restrictions on the availability (and/or on the use) of human data or samples. | NA |
| 15. Report the clinical trial registration number (at ClinicalTrials.gov or equivalent), where applicable. | NA |
| 16. For phase II and III randomized controlled trials, please refer to the CONSORT flow diagram (see link list at top right) and submit the CONSORT checklist (see link list at top right) with your submission. See author guidelines, under 'Reporting Guidelines'. Please confirm you have submitted this list. | NA |
| 17. For tumor marker prognostic studies, we recommend that you follow the REMARK reporting guidelines (see link list at top right). See author guidelines, under 'Reporting Guidelines'. Please confirm you have followed these guidelines. | NA |

## F- Data Accessibility

| | | |
|---|---|---|
| 18: Provide a "Data Availability" section at the end of the Materials & Methods, listing the accession codes for data generated in this study and deposited in a public database (e.g. RNA-Seq data: Gene Expression Omnibus GSE39462, Proteomics data: PRIDE PXD000208 etc.) Please refer to our author guidelines for 'Data Deposition'.<br><br>Data deposition in a public repository is mandatory for:<br>a. Protein, DNA and RNA sequences<br>b. Macromolecular structures<br>c. Crystallographic data for small molecules<br>d. Functional genomics data<br>e. Proteomics and molecular interactions | Raw mass spectrometry data was deposited within MassIVE (https://massive.ucsd.edu/ProteoSAFe/static/massive.jsp) as indicated within the data availability section. | |
| 19. Deposition is strongly recommended for any datasets that are central and integral to the study; please consider the journal's data policy. If no structured public repository exists for a given data type, we encourage the provision of datasets in the manuscript as a Supplementary Document (see author guidelines under 'Expanded View' or in unstructured repositories such as Dryad (see link list at top right) or Figshare (see link list at top right). | Source data are included within the extended view as datasets. | |
| 20. Access to human clinical and genomic datasets should be provided with as few restrictions as possible while respecting ethical obligations to the patients and relevant medical and legal issues. If practically possible and compatible with the individual consent agreement used in the study, such data should be deposited in one of the major public access-controlled repositories such as dbGAP (see link list at top right) or EGA (see link list at top right). | NA | |
| 21. Computational models that are central and integral to a study should be shared without restrictions and provided in a machine-readable form. The relevant accession numbers or links should be provided. When possible, standardized format (SBML, CellML) should be used instead of scripts (e.g. MATLAB). Authors are strongly encouraged to follow the MIRIAM guidelines (see link list at top right) and deposit their model in a public database such as Biomodels (see link list at top right) or JWS Online (see link list at top right). If computer source code is provided with the paper, it should be deposited in a public repository or included in supplementary information. | NA | The logistic curve fitting approach is sufficiently simple to be fully described in the "Logistic fitting of Spearman R values for hit prioritization" methods section |

## G- Dual use research of concern

| | |
|---|---|
| 22. Could your study fall under dual use research restrictions? Please check biosecurity documents (see link list at top right) and list of select agents and toxins (APHIS/CDC) (see link list at top right). According to our biosecurity guidelines, provide a statement only if it could. | NA |
